# Pathogenesis of Pulmonary Artery Remodeling: TGF-Beta Signaling and Inhibin Subunit Beta A in Group 1 and 2 Pulmonary Hypertension

**DOI:** 10.1161/ATVBAHA.125.322506

**Published:** 2026-01-22

**Authors:** Yusuke Yamada, Taijyu Satoh, Nobuhiro Yaoita, Kaito Yamada, Naoki Chiba, Kohei Komaru, Kotaro Nochioka, Saori Yamamoto, Haruka Sato, Nobuhiro Kikuchi, Takashi Nakata, Shinichiro Sunamura, Takumi Inoue, Hideka Hayashi, Hideaki Suzuki, Shunsuke Tatebe, Hiroyuki Takahama, Hisashi Oishi, Satoshi Miyata, Yoshinori Okada, Satoshi Yasuda

**Affiliations:** Department of Cardiovascular Medicine, Tohoku University Graduate School of Medicine, Sendai, Japan (Y.Y., T.S., N.Y., K.Y., N.C., K.K., K.N., S. Yamamoto, H. Sato, N.K., T.N., T.I., H.H., H. Suzuki, S.T., H.T., S. Yasuda).; Department of Medical Science and Innovation, SiRIUS Institute of Medical Research (T.S.), Tohoku University, Sendai, Japan.; Department of Thoracic Surgery, Institute of Development, Aging and Cancer (H.O., Y.O.), Tohoku University, Sendai, Japan.; Department of Cardiology, Sendai City Medical Center, Sendai Open Hospital, Japan (S.S.).; Teikyo University Graduate School of Public Health, Tokyo, Japan (S.M.).

**Keywords:** heart failure, hypertension, pulmonary, pulmonary artery, smooth muscle, transforming growth factors

## Abstract

**BACKGROUND:**

Pulmonary hypertension (PH) due to left heart disease (group 2 PH) is associated with a worse prognosis than isolated heart failure. Both pulmonary arterial hypertension (group 1 PH) and group 2 PH are involved in pulmonary artery (PA) remodeling, which is potentially driven by shared molecular mechanisms. The aim of this study was to investigate the underlying processes contributing to PA remodeling in group 2 PH.

**METHODS:**

To mimic the response to a left-sided pressure load, pulmonary arterial smooth muscle cells (PASMCs) were subjected to mechanical stretch. RNA sequencing of PAs from patients with group 2 PH was performed using the Gene Expression Omnibus database. Mice with transverse aortic constriction and spontaneously hypertensive rats were used as group 2 PH models, and they were treated with adeno-associated virus via intratracheal instillation.

**RESULTS:**

RNA sequencing of PASMCs after the stretch stress identified 1585 genes specifically upregulated in PASMCs from patients with group 1 PH. Further PA and plasma analyses from patients with group 2 PH, integrated with group 1 PH findings, identified enhancement of TGF-β (transforming growth factor-beta) signaling by the INHBA (inhibin subunit beta A) as a key feature. Metabolomics revealed that stretch-induced mitochondrial dysfunction in PASMCs caused lactic acidosis via enhancement of PDK1 (pyruvate dehydrogenase kinase 1) and c-MYC, leading to increased INHBA expression. Mice with transverse aortic constriction exhibited increased INHBA expression, decreased PDH (pyruvate dehydrogenase) expression, and acidic alterations in PAs. Targeted silencing of INHBA or PDK1 using adeno-associated virus in mice with transverse aortic constriction attenuated PA remodeling, improved right ventricular function, and reduced PH.

**CONCLUSIONS:**

Integrated RNA sequencing and metabolomics with stretched PASMCs and animal models identified mitochondrial dysfunction and subsequent acidic alterations as stimulators of increased INHBA expression and TGF-β signaling. These mechanisms contributed to PA remodeling in group 2 PH and provided potential therapeutic strategies.

**What Are the Clinical Implications?**

Integrated RNA sequencing and metabolomic analyses revealed that enhanced TGF-β (transforming growth factor-beta) signaling centered on INHBA (inhibin subunit beta A), which forms a homodimer to produce activin A, as well as mitochondrial dysfunction, are features shared by both group 1 and group 2 pulmonary hypertension (PH). In pulmonary arterial smooth muscle cells, mechanical stretch stimulation mimicking left-sided pressure overload induced metabolic dysfunction via the c-MYC-PDK1 (pyruvate dehydrogenase kinase 1) axis, leading to increased lactate production and subsequent cellular acidification. This acidic environment promoted the upregulation of INHBA expression and stimulated pulmonary arterial smooth muscle cell proliferation. These findings suggest a mechanism by which chronic biomechanical stress resulting from left heart failure drives vascular remodeling. Increased INHBA expression and metabolic dysfunction were observed in group 2 PH models, further supporting the pathogenic roles of these pathways. Targeted downregulation of INHBA and PDK1 expression in the lungs ameliorated pulmonary artery remodeling and improved PH in an experimental group 2 PH model. This indicates that INHBA contributes to disease progression. Taken together, these results demonstrate that INHBA-associated TGF-β signaling plays a critical role in the pathogenesis of group 2 PH and suggest that targeting INHBA may represent a promising therapeutic strategy for treating patients with group 2 PH.

Pulmonary hypertension (PH) due to left heart disease is classified as group 2 PH in the clinical classification of PH and is the most common type among patients with PH.^1^ Moreover, PH is present in 30% to 80% of patients with left heart failure.^1^ Recently, group 2 PH with elevated pulmonary vascular resistance (PVR) has been referred to as combined precapillary and postcapillary PH (CpcPH) and is associated with a poorer prognosis compared with isolated postcapillary PH, which does not involve an increase in PVR.^2–6^

As the 2022 European Society of Cardiology/European Respiratory Society characterized CpcPH as the mean pulmonary arterial (PA) pressure threshold at >20 mm Hg while reducing the PVR cutoff to >2 WU (Wood unit).^1^ The pathophysiology of CpcPH is considered to involve a greater degree of pulmonary vascular disease compared with Ipc-PH.^7^ However, research on the pulmonary vasculature in group 2 PH has been limited by restricted access to appropriate lung samples and marked clinical variability among patients. These challenges have made it difficult to examine the cellular mechanisms underlying the disease. Because patients with CpcPH experience unfavorable outcomes, a clearer understanding of the pulmonary vascular component remains an important objective, and efforts to outline an initial mechanistic framework may help stimulate further investigation.

Group 1 PH (PA hypertension [PAH]) and group 2 PH, particularly CpcPH, are distinct clinical entities. Nevertheless, several studies have reported overlap in selected aspects of pulmonary vascular remodeling between these conditions. In particular, CpcPH and PAH have been shown to share certain hemodynamic characteristics and pulmonary vascular features, as well as histopathologic findings such as medial hypertrophy, fibrosis, and luminal occlusion of distal PAs.^8–11^ At the cellular level, PA smooth muscle cells (PASMCs) derived from patients with group 2 PH have been reported to exhibit enhanced proliferative activity, a feature also described in PAH.^12^ In addition, genetic analyses by Assad et al^13^ identified shared susceptibility loci related to metabolic pathways and angiogenesis, suggesting partial molecular overlap. Transcriptomic studies further demonstrated similarities in PA endothelial cell (PAEC) gene expression profiles between patients with PAH and CpcPH,^14^ and metabolomic analyses have reported overlapping alterations, including reduced levels of prostaglandins and nitric oxide–related metabolites such as linoleic acid, arginine, and homoarginine.^15^ Importantly, these observations do not indicate mechanistic equivalence between group 1 and group 2 PH. Rather, they suggest that certain cellular and molecular pathways relevant to pulmonary vascular remodeling may intersect. Clarifying which components are shared at a pathway or cellular level, and which remain specific to GROUP 2 PH, may provide a contextual framework for focused mechanistic investigation.

In addition, from a clinical perspective, comparisons between CpcPH and isolated postcapillary PH have shown no significant differences in PA wedge pressure or cardiac index.^16^ Interestingly, some reports have indicated smaller left ventricular and left atrial diameters in CpcPH,^13,17^ suggesting that factors other than elevated left-sided pressure or the severity of left heart disease may contribute to pulmonary vascular remodeling and increased PVR.^18^ The 2-hit hypothesis indicated that sustained elevated left-sided pressure, combined with additional triggers, drives PA remodeling in group 2 PH.^7^ However, the specific molecular mechanisms underlying these processes remain unclear.

INHBA (inhibin subunit beta A), a component of activin and inhibin, regulates stromal cell proliferation and has been implicated in PH.^19^ Dysregulated BMP (bone morphogenetic protein) and maladaptive TGF-β (transforming growth factor-beta) signaling contribute to PA remodeling, while activin is a ligand in TGF-β pathways.^20^ Sotatercept, targeting TGF-β signaling, is a promising treatment for group 1 PH.^21–23^ However, despite these advances, INHBA’s role and regulation in group 2 PH pathogenesis remains unknown despite these advances.

Therefore, in this study, we aimed to investigate the mechanisms underlying PA remodeling in response to left-sided pressure load and the shared features of groups 1 and 2 PH. To model pressure overload, PASMCs subjected to mechanical stretch and PA data from patients with group 2 PH were analyzed using the National Center for Biotechnology Information Gene Expression Omnibus (GEO) database (GSE236251).^24^ Combined RNA sequencing and metabolomics analyses were conducted to identify the shared mechanisms. In addition, group 2 PH in vivo models were genetically modified to analyze INHBA’s role.

## Methods

### Data Availability

The data underlying this article will be shared on reasonable request to the corresponding author.

### Clinical Data

All protocols were approved by the institutional review board of Tohoku University, Sendai, Japan, and complied with the ethical guidelines of the Declaration of Helsinki. Before this study, we obtained written informed consent from the patients for conducting a case-control study or a retrospective cohort study. For secondary research use of plasma samples, the medical ethics review committee has approved to waive written informed consent by using the opt-out method (Approval numbers, Plasma samples; 2021-1-208). The data of patients were collected on the date of each preservation (Table S1), not including their prognosis. Reported data follow the STROBE (the Strengthening the Reporting of Observational Studies in Epidemiology) initiative.

We conducted a retrospective investigation involving 81 patients with group 2 PH who had undergone right heart catheterization between January 2021 and July 2025 in Japan. The patients were sequentially enrolled at Tohoku University Hospital in Japan. All samples were utilized to evaluate the plasma level of activin A, follistatin, and FSTL3 (follistatin-like 3). The diagnosis was established through a combination of echocardiography, computed tomography, spirometry, ventilation/perfusion lung scans, and right heart catheter examination, adhering to the guidelines established by the European Society of Cardiology and the European Respiratory Society in 2015 and 2022, respectively.^4,12^ Diagnostic criteria included a resting mean PA pressure of ≥20 mm Hg, as measured via right cardiac catheterization, and a PA wedge pressure of >15 mm Hg. Exclusion criteria were as follows: patients with precapillary PH. Plasma samples were collected from the PA using a Swan-Ganz catheter.

### Human Lung Samples

We obtained lung tissues from patients with PAH during lung transplantation (Table S2) and from nonpatients with PH undergoing thoracic surgery for lung cancer. Tissue samples from the latter were collected from areas distant from the tumor margins, as previously documented.^25^ All study protocols met the ethical guidelines of the Declaration of Helsinki and were approved by the institutional review board of Tohoku University, Sendai, Japan. We obtained written informed consent from patients with PH in case-control or prior studies. The medical ethics review committee approved the use of an opt-out method or secondary research using lung tissues, which waived the requirement for written informed consent (Approval number: 2021-1-1076). PASMCs and PAECs with an outer diameter of <1.5 mm were isolated from PAs after established procedures.^25^ The cells were cultured in DMEM (Thermo Fisher Scientific, Waltham) supplemented with 10% fetal bovine serum, and PAECs were cultured in endothelial cell basal medium containing endothelial growth supplement (Promo Cell, Heidelberg, Germany). They were maintained at 37 °C in a humidified atmosphere containing 5% CO_2_ and 95% air, respectively. PASMCs and PAECs at passages 4 to 7, reaching 70% to 80% confluence, were used in the experiments.

### Animal Experiments

All animal experiments were conducted according to the protocols approved by the Tohoku University Animal Care and Use Committee (Approval no. 2022-ido-036) and were compliant with Regulations for Animal Experiments and Related Activities at Tohoku University and the ARRIVE (Animal Research: Reporting In Vivo Experiments) guidelines. Male mice were selected for this study to avoid potential influences of sex hormones on the results. Experiments were performed on 10-week-old male C57BL/6J mice and 18-week-old male spontaneously hypertensive rats or control Wistar Kyoto rats (Japan SLC, Shizuoka, Japan). Mice were randomly assigned to different experimental groups by alternately allocating them to each group. Transverse aortic constriction (TAC) was performed as described.^26^ We anesthetized the mice briefly with isoflurane and maintained their temperature throughout the procedure. The transverse aorta was constricted using a 6-0 suture tied tightly around a 27-gauge needle, which was subsequently removed. The sham-operated mice underwent the same procedure without ligating the transverse aorta. In this study, we included mice with a transverse aortic velocity exceeding 4 m/s, as assessed by echocardiography. Heart failure assessment and histology were conducted 4 weeks after surgery. Furthermore, we measured the right ventricular systolic pressure (RVSP), right ventricular hypertrophy, and pulmonary vascular remodeling to evaluate the development of PH in group 2. A 1.2-F pressure catheter (SciSense Inc., Ontario, Canada) was inserted through the right jugular vein into the right ventricle during right heart catheterization to measure the RVSP. The PowerLab data acquisition system (AD Instruments, Bella Vista, Australia) was used to record and analyze the data with values averaged over 10 consecutive beats. Finally, all mice and rats were deeply anesthetized with isoflurane (>4.5%) and euthanized.^27^

### Statistical Analysis

All plots were composed of 3 to 12 independent biological replicates, and results are expressed as mean±SD or median and interquartile range (IQR). The Mann-Whitney *U* test was used to compare 2 groups. The Kruskal-Wallis test, alongside Dunn multiple comparison tests, was used for comparisons between 3 or more groups. A 2-way ANOVA was used to analyze the mean responses associated with the 2 main effects of the distinct groups, followed by the Tukey honest significant difference test for multiple comparisons. Statistical analyses, including Pearson’s correlation,^28,29^ were performed using GraphPad Prism 9 (GraphPad Software, San Diego, CA). Statistical significance was set at *P*<0.05 (2-tailed).^30,31^ All experiments were conducted by different researchers in a blinded manner.

## Results

### INHBA as a Key Contributor to PA Remodeling in Group 1 and Group 2 PH

**Figure 1.**
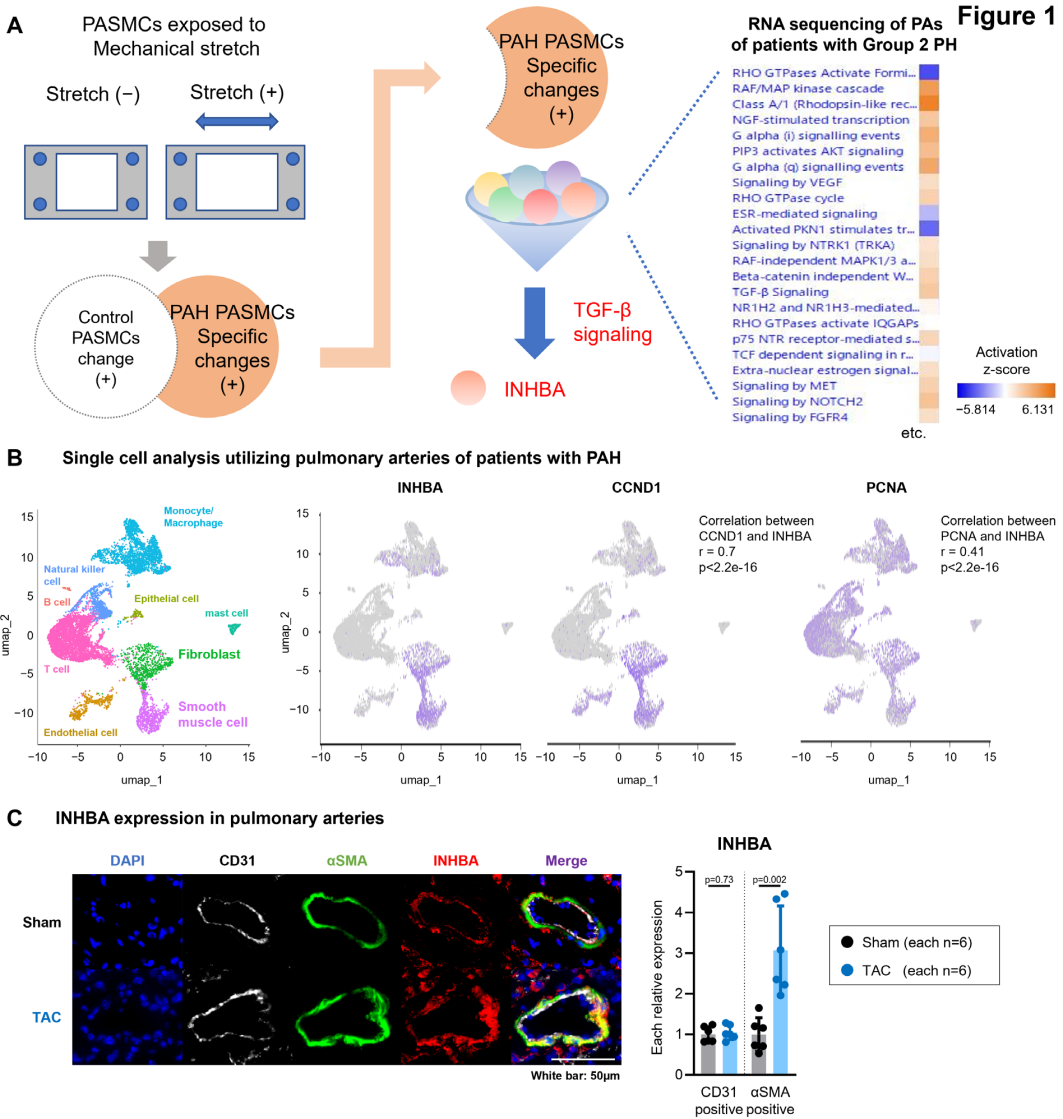
**Stretch-induced activation of TGF-β (transforming growth factor-beta) signaling with INHBA (inhibin subunit beta A) as a central regulator in group 1 and group 2 pulmonary hypertension (PH). A**, Schematic illustration of RNA sequencing using cultured pulmonary arterial smooth muscle cells (PASMCs) subjected to a 24-hour stretch from patients with pulmonary arterial hypertension (PAH) and individuals without PH. Additional RNA sequencing data from pulmonary arteries of patients with group 2 PH were analyzed using the Gene Expression Omnibus (GEO) data sets. First, we confirmed factors that were specifically increased in PAH PASMCs exposed to mechanical stretch. In addition, GEO analyses of pulmonary arteries revealed mechanisms involved in cell proliferation, including TGF-beta signaling. Notably, the increase in INHBA expression, which is involved in TGF-beta signaling, was consistently observed in both the mechanical stretch and GEO analyses, supporting the focus on activin A signaling in our study. **B**, UMAP projections of human pulmonary artery (PAH) single-cell data from the National Center for Biotechnology Information GEO database (GSE228644), showing feature plots of INHBA, PCNA (proliferating cell nuclear antigen), and CCND1 (cyclin D1) expression. **C**, Immunofluorescence images of pulmonary arteries from transverse aortic constriction (TAC) mice and sham control, showing αSMA (smooth muscle actin alpha; green), INHBA (red), CD31 (white), and DAPI (4',6-diamidino-2-phenylindole; blue) staining, followed by relative expression of INHBA in smooth muscle or endothelial cells. Data are presented as mean±SD and were analyzed using the Mann-Whitney *U* test. AKT indicates AKT serine/threonine kinase; CD31, PECAM-1 (platelet endothelial cell adhesion molecule-1); ESR, estrogen receptor; FGFR4, fibroblast growth factor receptor 4; MAP, mitogen-activated protein kinase; MET, MET proto-oncogene; NGF, nerve growth factor; NOTCH2, notch receptor 2; NR1H, nuclear receptor subfamily 1 group H; NTRK1, neurotrophic receptor tyrosine kinase 1; p75NTR, NGF receptor; PIP3, phosphatidylinositol 3,4,5-trisphosphate; PKN1, protein kinase N1; RAF, rapidly accelerated fibrosarcoma; TCF, T-cell factor; TRKA, tropomyosin-related kinase A; UMAP, uniform manifold approximation and projection; and VEGF, vascular endothelial growth factor.

The study initially focused on stretch-induced alterations in PASMCs to identify specific changes associated with PAH (Figure 1A and 1B). RNA sequencing analysis of PAs from patients with group 2 PH (GEO database [GSE236251])^24^ highlighted TGF-β signaling, with INHBA as a major component (Figure 1A). Single-cell analysis of PAs from patients with PAH (GEO database [GSE228644])^32^ demonstrated INHBA expression predominantly in smooth muscle cells, which was colocalized with CCND1 (cyclin D1) and proliferating cell nuclear antigen (Figure 1B). These findings were validated by a statistically significant correlation between their expression levels (Figure 1B; Figure S1A).

Next, we performed a case-control study on patients with group 2 PH and examined plasma samples to identify potential clinical biomarkers. Patients with group 2 PH associated with PVR >2 WU showed lower prevalence of male (PVR ≤2 WU, n=37 versus PVR >2 WU, n=44; 81% versus 41%; *P*=0.0002), and higher mean PA pressure (median 25 [IQR, 22–29] versus 29 [IQR, 24–35] mm Hg; *P*=0.0049; Table S1). Plasma activin A levels, a homodimer of INHBA, were significantly higher in patients with group 2 PH with PVR >2 WU compared with those with PVR ≤2 WU (PVR ≤2 WU, n=37 versus PVR >2 WU, n=44; median 249.0 [IQR, 144.4–465.9] versus 514.7 [IQR, 207.3–2482.0] pg/mL; *P*=0.02; Figure S1B). Follistatin levels, which increase in response to activin A to counteract activin A, were also higher in patients with PVR >2 WU (median 1763 [IQR, 1227–2321] versus 2037 [IQR, 1535–2743] pg/mL; *P*=0.04). FSTL3 plasma level, one of the endogenous antagonists against activin, was increased (median 6.48 [IQR, 4.79–11.03] versus 9.03 [IQR, 5.75–12.13] ng/mL; *P*=0.06). Activin A and follistatin or FSTL3 levels were positively correlated (follistatin: *r*=0.561, *P*<0.001, FSTL3: *r*=0.2938; *P*=0.0082), supporting the possibility of a primary increase in INHBA expression (Figure S1C).

Elevated INHBA expression was observed in the PAs of group 2 PH animal models, including TAC mice and spontaneously hypertensive rats (Figures S2, S3A, and S3B), consistent with our findings (Figure 1C; Figure S3C). This upregulation was associated with PA remodeling compared with respective controls (Figure 1C; Figure S3C).

### Stretch-Induced INHBA Expression Promotes Cell Proliferation and TGF-β Signaling in PASMCs

**Figure 2.**
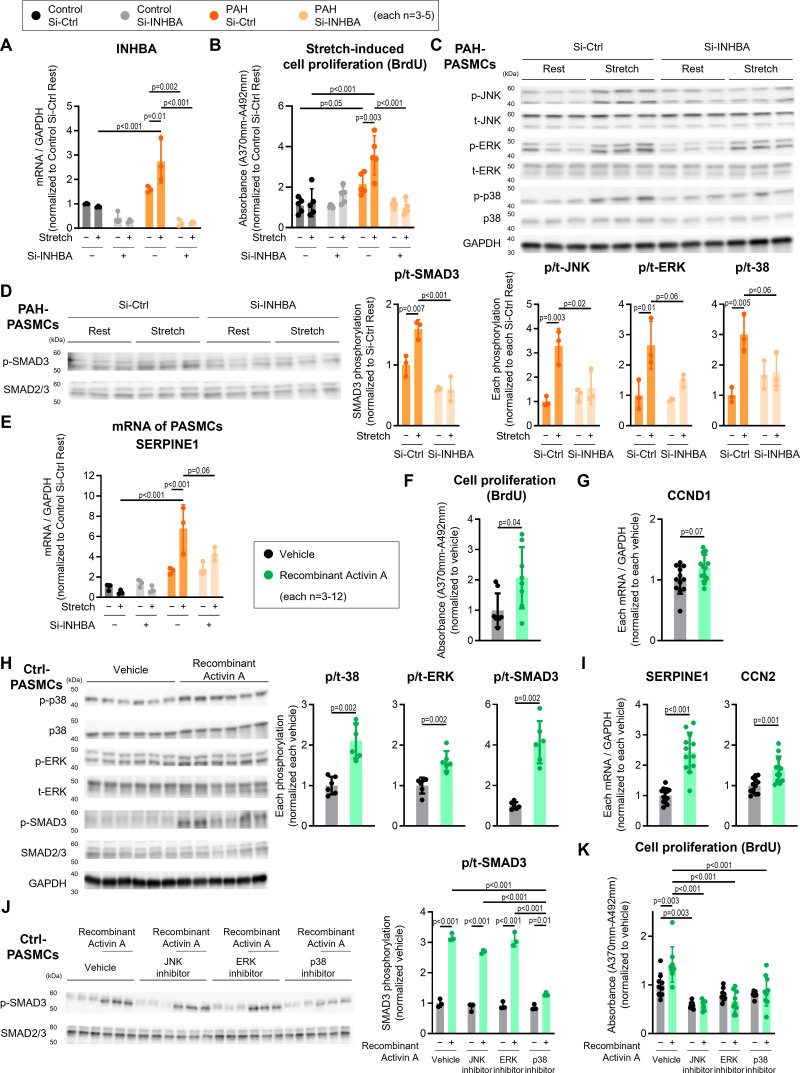
**INHBA (inhibin subunit beta A) regulates stretch-induced cell proliferation in pulmonary arterial hypertension (PAH) pulmonary arterial smooth muscle cells (PASMCs). A**, mRNA expression levels of INHBA and (**B**) 5-bromo-2′-deoxyuridine (BrdU) incorporation assays showing cell proliferation in PASMCs from individuals without pulmonary hypertension (PH) and individuals with PAH stretched for 24 hours, with si-INHBA or si-Ctrl treatment (n=3). **C**, Representative Western blots and quantification of p-JNK (phosphorylated c-Jun N-terminal), t-JNK (total JNK), p-ERK (phosphorylated extracellular signal–regulated kinase), t-ERK (total Erk), p-p38 (phosphorylated p38 mitogen-activated protein kinase), t-p38 (total-p38), and GAPDH expression, (**D**) p-SMAD3 (phosphorylated SMAD3) and t-SMAD3 (total-SMAD3) expression and (**E**) mRNA expression levels of SERPINE1 (serpin family E member 1) in PASMCs from individuals with PAH stretched for 24 hours and transfected with si-INHBA or si-Ctrl (n=3). **F**, BrdU incorporation assays showing cell proliferation in cultured PASMCs from individuals without PH treated with recombinant activin A (100 ng/mL) for 24 hours (n=8). **G**, mRNA expression levels of CCND1 (cyclin D1) in cultured PASMCs from individuals without PH treated with recombinant activin A for 6 hours (n=8). **H**, Representative Western blots and quantification of p-p38, t-p38, p-ERK, t-ERK, p-SMAD3, t-SMAD3, and GAPDH expression in PASMCs treated with recombinant activin A for 24 hours (n=6). **I**, mRNA expression levels of SERPINE1 and CCN2 (cellular communication network factor 2) in cultured PASMCs from individuals without PH treated with recombinant activin A for 6 hours (n=12). **J**, Representative Western blots and quantification of p-SMAD3 and t-SMAD3 expression in PASMCs and (**K**) cell proliferation of PASMCs from individuals without PH treated with recombinant activin A (100 ng/mL) or JNK inhibitor (SP600125, 20 μM), ERK inhibitor (PD98059, 20 μM), or p-p38 inhibitor (SB203580, 10 μM). Data are presented as mean±SD, and (**A** through **E**, **J**, and **K**) were analyzed using 2-way ANOVA followed by Tukey honest significant difference test or (**F** through **I**) the Mann-Whitney *U* test. Ctrl indicates Control; Si-Ctrl, small interfering control; and si-INHBA, small interfering INHBA.

Mechanical stretch-induced increased INHBA mRNA expression in PASMCs derived from patients with PAH (Figure 2A). In contrast, no significant changes were noted in INHBA expression in PAECs under similar conditions (Figure S4A). In vivo, INHBA expression was detected in the CD31 (PECAM-1; platelet endothelial cell adhesion molecule-1)-positive endothelial cell layer of PAs by immunofluorescence. Although INHBA-positive signals were observed in endothelial cells, no consistent or marked difference between TAC and sham mice was evident by qualitative image assessment (Figure 1C). To further examine endothelial INHBA expression, quantitative analysis using flow cytometry was performed. INHBA mRNA expression in isolated PAECs (CD45⁻/CD326⁻/CD31⁺ cells) from TAC mice was comparable to that in sham mice, suggesting that the pronounced overall upregulation of INHBA observed in PAs is unlikely to be primarily attributable to the endothelial layer (Figure S4B). In contrast, INHBA expression was significantly increased in the smooth muscle layer of TAC mice (Figure 1C), suggesting that smooth muscle–derived INHBA plays a predominant role in the development of group 2 PH.

To further evaluate the potential contribution of endothelial-to-mesenchymal transition, PAECs were incubated with recombinant activin A for 1 to 7 days. No statistically significant changes were observed in the expression of mesenchymal markers (S100A4, ACTA2 [actin alpha 2], TAGLN [Transgelin], Snail1, and Snail2) or endothelial markers (VE-cadherin [vascular endothelial cadherin] and PECAM1 [platelet endothelial cell adhesion molecule-1]) at either the mRNA or protein levels (Figure S4C and S4D). In addition, immunofluorescence analysis showed no appreciable change in VE-cadherin expression after activin A treatment (Figure S4E). These results suggest that activin A stimulation does not significantly induce endothelial-to-mesenchymal transition and may not play a major role in PA remodeling in the group 2 PH model.

INHBA expression was selectively downregulated in PASMCs using specific small interfering RNA (siRNA) to investigate its role, which effectively reduced both baseline- and stretch-induced INHBA expression (Figure 2A). Cell proliferation was assessed by 5-bromo-2′-deoxyuridine incorporation. PAH PASMCs exhibited higher proliferation at baseline than the control PASMCs, with increased enhancement under stretch stress (Figure 2B). Downregulation of INHBA using siRNA attenuated stretch-induced proliferation (Figure 2B). Regarding the involvement of signaling pathways, stretch stress increased the phosphorylation of JNK (c-Jun N-terminal), ERK (extracellular signal–regulated kinase), p38 (p38 mitogen-activated protein kinase), and SMAD3 in association with TGF-β, which was partially mitigated by siRNA-mediated INHBA downregulation (Figure 2C and D). However, the attenuation was not complete. Similarly, SERPINE1 (serpin family E member 1) mRNA expression, a downstream target of TGF-β signaling, showed a comparable pattern (Figure 2E). These data indicated that stretch-induced alteration of JNK, ERK, p38, and TGF-β signaling in PASMCs mainly occurs via INHBA.

PASMCs were treated with recombinant activin A (100 ng/mL) for 24 hours to elucidate the role of INHBA. This stimulation significantly increased the proliferation of the control PASMCs (Figure 2F) and was associated with elevated CCND1 expression (Figure 2G). Furthermore, recombinant activin A enhanced the phosphorylation of components involved in the mitogen-activated protein kinase pathways, including p38, ERK, and SMAD3 (Figure 2H), and upregulated mRNA levels of SERPINE1, CCN2 (cellular communication network factor 2; Figure 2I). To delve into this mechanism, control PASMCs were incubated with activin A and JNK (SP600125, 20 μM), ERK (PD98059, 20 μM), or p-p38 inhibitor (SB 203580, 10 μM). The p38 inhibitor attenuated the activin A–induced increase in SMAD3 phosphorylation (Figure 2J), supporting the involvement of p38 in this process. In contrast, JNK and ERK inhibition had no effect, suggesting that activin A–induced SMAD3 activation was mediated specifically through p38. Consistent with these findings, JNK, ERK, or p38 inhibition markedly suppressed activin A–induced PASMC proliferation (Figure 2K). These findings support the hypothesis that the stretch-induced activin A–SMAD3 axis is largely dependent on p38 signaling.

### Metabolic Dysfunction in PAH PASMCs Enhances the Warburg Effect, Thereby Inducing Acidosis and INHBA Expression Under Stretch

**Figure 3.**
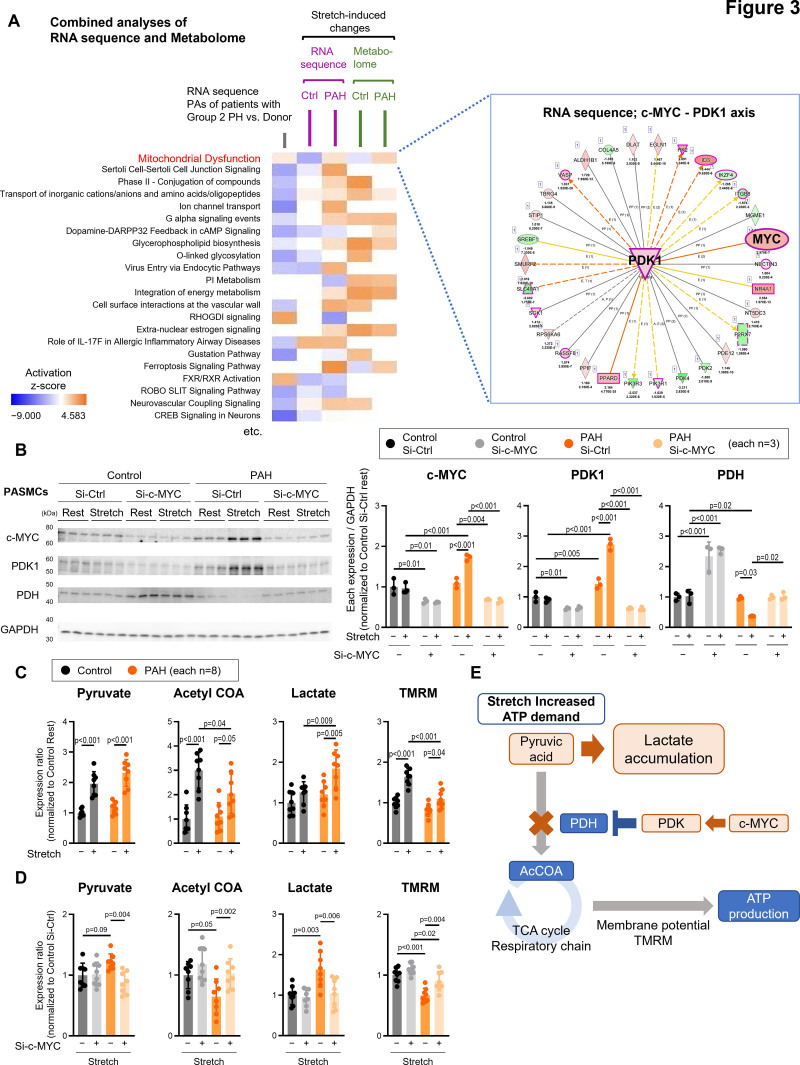
**Metabolic dysfunction exacerbates lactic acidosis in pulmonary arterial hypertension (PAH) pulmonary arterial smooth muscle cells (PASMCs). A**, Heatmap of RNA sequencing data from pulmonary arteries (PAs) of patients with group 2 pulmonary hypertension (PH), combined with a heatmap of stretch-induced changes analyzed via RNA sequencing and metabolomics in cultured control and PAH PASMCs. **B**, Representative Western blots and quantification of c-MYC, PDK1 (pyruvate dehydrogenase kinase 1), PDH (pyruvate dehydrogenase), and GAPDH expression in PASMCs from patients with PAH and control individuals (n=3). **C**, Quantification of pyruvate, acetyl-CoA, lactate, and tetramethylrhodamine methyl ester (TMRM) in PASMCs from patients with PAH and control individuals subjected to stretch for 24 hours (n=8). **D**, Quantification of pyruvate, acetyl-CoA (coenzyme A), lactate, and TMRM in PASMCs from patients with PAH and control individuals stretched for 24 hours and transfected with si-c-MYC or si-Ctrl (n=8). **E**, Schematic representation of stretch-induced lactate accumulation specific to PASMCs from patients with PAH subjected to 24 hours of stretch. Data are presented as mean±SD. Comparisons between each group were analyzed using 2-way ANOVA followed by Tukey honest significant difference test. AcCoA indicates acetyl-coenzyme A; cAMP, cyclic adenosine monophosphate; CREB, cAMP response element-binding protein; Ctrl, Control; DARPP32, dopamine and cAMP-regulated phosphoprotein 32 kDa; FXR, farnesoid X receptor; IL, interferon; PI, phosphoinositide; RHOGDI, rho guanine nucleotide dissociation inhibitor; ROBO, roundabout; RXR, retinoid X receptor; si-c-MYC, small interfering c-MYC; si-Ctrl, small interfering control; and TCA, tricarboxylic acid.

Comprehensive analyses using RNA sequencing and metabolomics revealed mitochondrial dysfunction in the PAs of both group 2 PH and stretched PAH PASMCs (Figure 3A). Ingenuity pathway analysis of stretch-induced changes showed activation of the c-MYC–PDK1 (pyruvate dehydrogenase kinase 1) axis in PAH PASMCs (Figure 3A). Stretching increased c-MYC and PDK1 expression and decreased PDH (pyruvate dehydrogenase) in PAH PASMCs at the protein level, which affected pyruvate utilization and the tricarboxylic acid cycle (Figure 3B and 3E). These alterations were attenuated by the siRNA-mediated downregulation of c-MYC (Figure 3B).

Metabolomics confirmed that the stretch stress promoted glucose metabolism in both PAH and non-PH PASMCs (Figure S5). Further investigation into mitochondrial energy metabolism revealed that mechanical stretch increased pyruvate, acetyl-CoA (coenzyme A), and tetramethylrhodamine methyl ester levels, suggesting activation of the tricarboxylic acid cycle (Figure 3C and 3E). However, the stretch-induced increases in acetyl-CoA and tetramethylrhodamine methyl ester were attenuated in PAH PASMCs, likely due to impaired utilization of acetyl-CoA as a substrate for the tricarboxylic acid cycle via upregulation of the c-MYC–PDK–PDH axis. Consistent with this, stretch-induced lactate accumulation was observed specifically in PAH PASMCs (Figure 3C), supporting the enhancement of the Warburg effect under these conditions (Figure 3E). Moreover, siRNA-mediated downregulation of c-MYC reduced lactate production and increased acetyl-CoA and tetramethylrhodamine methyl ester levels (Figure 3D), indicating that c-MYC regulates glucose metabolism in PAH PASMCs in response to mechanical stretching (Figure 3E).

**Figure 4.**
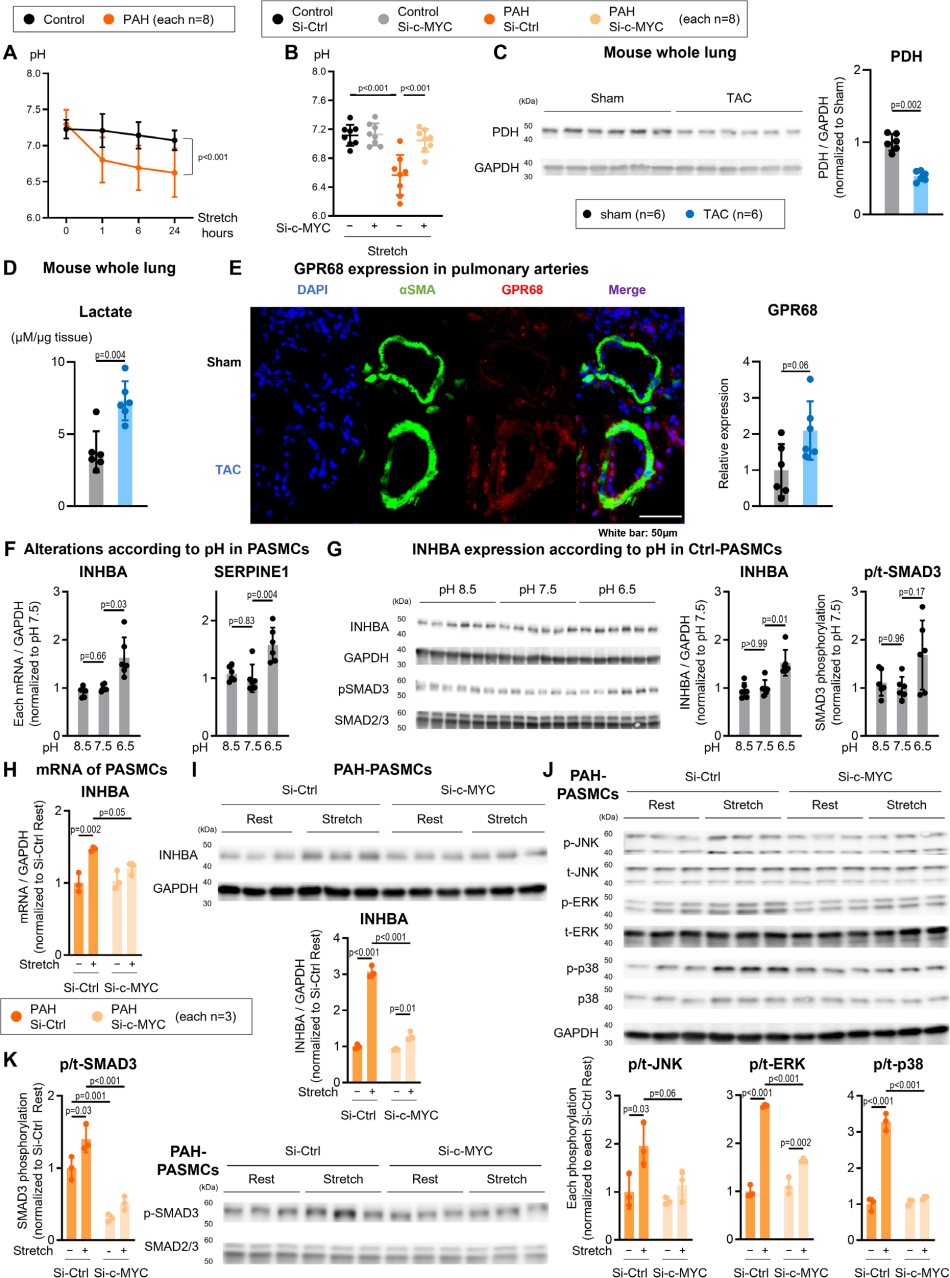
**Acidosis in stretched pulmonary arterial smooth muscle cells (PASMCs) and pulmonary arteries of group 2 pulmonary hypertension (PH) animal models. A**, pH measurements in PASMCs from patients with pulmonary arterial hypertension (PAH) and individuals without PH subjected to stretch for 0, 1, 6, and 24 hours. **B**, pH measurements in PASMCs from individuals without PH and individuals with PAH stretched for 24 hours and transfected with si-c-MYC or si-Ctrl. **C**, Representative Western blots and quantification of PDH (pyruvate dehydrogenase) and GAPDH expression in the whole lungs from transverse aortic constriction (TAC) mice. **D**, Lactate levels of the whole lung from TAC mice. **E**, Representative images and quantification of αSMA (smooth muscle actin alpha; green), GPR68 (G-protein–coupled receptor 68; red), and DAPI (4',6-diamidino-2-phenylindole; blue) expression in the pulmonary arteries of TAC mice and sham control. **F**, mRNA expression of INHBA (inhibin subunit beta A) and SERPINE1 (serpin family E member 1), and (**G**) representative Western blots and quantification of INHBA and GAPDH or p-SMAD3 (phosphorylated SMAD3) or SMAD3 expression in cultured PASMCs from individuals without PH incubated in DMEM with adjustment of pH to 8.5, 7.5, or 6.5. **H**, mRNA expression levels of INHBA in PASMCs from individuals with PAH stretched for 24 hours, with si-c-MYC or si-Ctrl treatment (n=3). **I**, Representative Western blots and quantification of INHBA and GAPDH in PASMCs from individuals with PAH stretched for 24 hours, with si-c-MYC or si-Ctrl treatment (n=3). **J**, Representative Western blots and quantification of p-JNK (phosphorylated c-Jun N-terminal), t-JNK (total JNK), p-ERK (phosphorylated extracellular signal–regulated kinase), t-ERK (total ERK), p-p38 (phosphorylated p38 mitogen-activated protein kinase), t-p38 (total-p38), and GAPDH expression in PASMCs from individuals with PAH stretched for 24 hours and transfected with si-c-MYC or si-Ctrl. **K**, Representative Western blots and quantification of p-SMAD3 and t-SMAD (total-SMAD3) in PASMCs from individuals with PAH stretched for 24 hours, with si-c-MYC or si-Ctrl treatment (n=3). Data are presented as mean±SD, and (**A**) were analyzed using repeated measures ANOVA with the Geisser-Greenhouse correction, **B** and **H** through **K** were analyzed using 2-way ANOVA followed by Tukey honest significant difference test, (**C** through **E**) using the Mann-Whitney *U* test, or (**F** and **G**) using the Kruskal-Wallis test followed by Dunn test. Ctrl indicates Control; si-c-MYC, small interfering c-MYC; and si-Ctrl, small interfering control.

Stretch-induced lactate accumulation led to acidic conditions in PAH PASMCs (Figure 4A), which were attenuated by *c-MYC* knockdown using siRNA (Figure 4B). Consistent with the in vitro findings, PDH expression was reduced in the lungs of TAC mice and spontaneously hypertensive rats (Figure 4C; Figure S6A), accompanied by elevated lactate levels (Figure 4D). Furthermore, PAs from TAC mice and spontaneously hypertensive rats, likely subjected to pressure overload, showed increased expressions of GPR68 (G-protein–coupled receptor 68), consistent with a lower pH environment (Figure 4C; Figure S6B). These results support the contribution of metabolic dysfunction to an acidic environment in the pulmonary vasculature.

Crystal violet staining was performed to evaluate cell viability in vitro conditions, which demonstrated that some PASMCs died at pH 5.5 compared with pH 6.5 or 7.5 (Figure S6C). To accurately evaluate expression changes, analyses were focused on pH conditions ranging from 8.5 to 6.5. At pH 6.5, INHBA expression was upregulated at both the mRNA and protein levels compared with pH 7.5, alongside an increase in SERPINE1 expression and phosphorylation of SMAD3 (Figure 4F and 4G). These findings suggest that acidic conditions enhance TGF-β signaling in PASMCs.

To clarify the role of c-MYC, a key regulator of energy metabolism, we downregulated its expression in PASMCs exposed to mechanical stretch using siRNA. This intervention substantially suppressed the stretch-induced upregulation of INHBA and reduced phosphorylation of ERK, JNK, p-p38, and SMAD3 (Figure 4H through 4K). However, because these effects were not completely abolished, additional stretch-responsive mechanisms are likely involved in regulating both INHBA expression and downstream signaling. These results support the hypothesis that p38 signaling is primarily involved in TGF-β activation via INHBA, whereas INHBA and c-MYC also regulate JNK and ERK pathways independently (Figure S7).

These findings imply that PAH PASMCs exhibit impaired responses to mechanical stress, caused by mitochondrial dysfunction at the core of glucose metabolism. Mechanical stretch results in lactic acidosis, which contributes to cell proliferation and PA remodeling through increased INHBA (Graphical Abstract).

### Role of INHBA in PA Remodeling in TAC Mice

**Figure 5.**
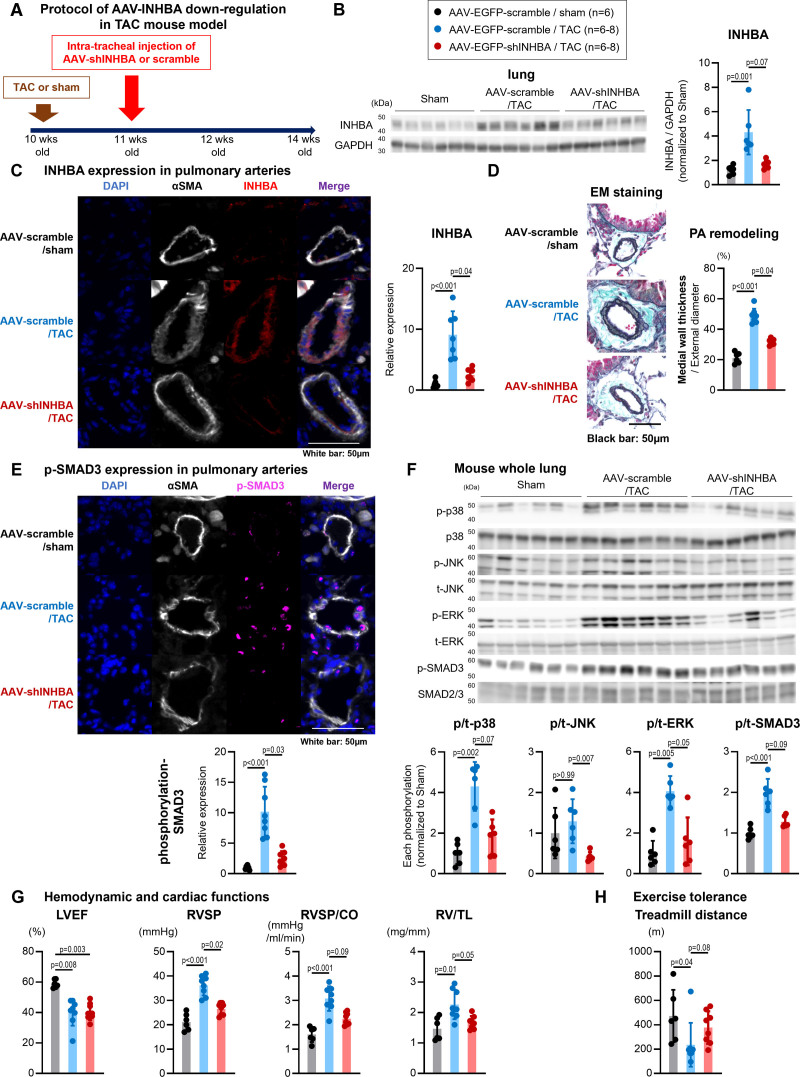
**Inhibition of INHBA (inhibin subunit beta A) via adeno-associated virus (AAV) transduction attenuates pulmonary artery remodeling and cell proliferation signaling in transverse aortic constriction (TAC) mice. A**, Experimental timeline for the inhibition of INHBA signaling through AAV transduction via intratracheal instillation in TAC mice. **B**, Western blots and quantification of INHBA and GAPDH expression in lung tissue collected from TAC mice treated with AAV (n=6). **C**, Representative immunofluorescence images and quantification of αSMA (smooth muscle actin alpha; white), INHBA (red), and DAPI (4',6-diamidino-2-phenylindole; blue) expression in distal pulmonary arteries (PAs) of TAC mice. Scale bar=50 µm. **D**, Representative Elastica Masson (EM) images and quantification of pulmonary artery remodeling in TAC mice treated with AAV. **E**, Representative immunofluorescence images and quantification of αSMA (white), p-SMAD3 (phosphorylated SMAD3; purple), and DAPI (blue) expression in distal pulmonary arteries (PAs) of TAC mice. Scale bar=50 µm. **F**, Western blots and quantification of p-p38 (phosphorylated p38 mitogen-activated protein kinase), t-p38 (total p38), p-JNK (phosphorylated c-Jun N-terminal), t-JNK (total JNK), p-ERK (phosphorylated extracellular signal–regulated kinase), t-ERK (total ERK), p-SMAD3, and t-SMAD3 (total SMAD3) in lung samples from TAC mice treated with AAV (n=6). **G**, Echocardiographic measurements assessing left ventricular ejection fraction (LVEF), right ventricular systolic pressure (RVSP), RVSP/ cardiac output (CO) ratio (total pulmonary resistance), and the ratio of right ventricular weight to tibia length (RV/TL) in TAC mice treated with AAV (n=6–8). **H**, Treadmill test performance of TAC mice treated with AAV (n=6–8). Data are presented as mean±SD. Comparisons between each group were analyzed using the Kruskal-Wallis test followed by Dunn test. AAV-shINHBA indicates adeno-associated virus-short hairpin INHBA; and EGFP, enhanced green fluorescent protein.

To assess whether attenuation of INHBA can mitigate PA remodeling and PH in group 2 PH in vivo, we utilized a recombinant AAV carrying EGFP under the cytomegalovirus promoter to downregulate INHBA (AAV6-shINHBA [short hairpin INHBA]) in PAs (Figure 5A; Figure S8A).^27,33^ AAV6-shINHBA was administered intratracheally 1 week after performing TAC in 10-week-old C57BL/6J mice. Three weeks later, we harvested lung tissues to evaluate INHBA expression. This approach significantly reduced INHBA levels in lung homogenates and in the PA smooth muscle layer (Figure 5B and 5C). Elastica Masson staining showed that INHBA downregulation attenuated PA remodeling (Figure 5D). Increased phosphorylation of SMAD3, p38, JNK, and ERK was observed in the PAs in mice with TAC, which was attenuated by AAV6-shINHBA administration (Figure 5E and 5F). Hemodynamic analysis revealed that TAC mice exhibited decreased left ventricular ejection fraction, increased RVSP, total pulmonary resistance (RVSP/cardiac output), and right ventricular weight to tibia length ratio (Figure 5G; Figure S8B), with no effect of blood pressure and heart rate (Figure S8B). However, a reduction in INHBA expression restored RVSP and total pulmonary resistance and improved right ventricular function, including parameters such as pulmonary acceleration time, tricuspid annular plane systolic excursion, right ventricular fractional area change (Figure S9), and treadmill exercise tolerance (Figure 5H). No effects of AAV6-shINHBA were noted on hemoglobin levels (Figure S8B).

### Energy Metabolism Centered on the PDK1-PDH Axis in the PA of TAC Mice

**Figure 6.**
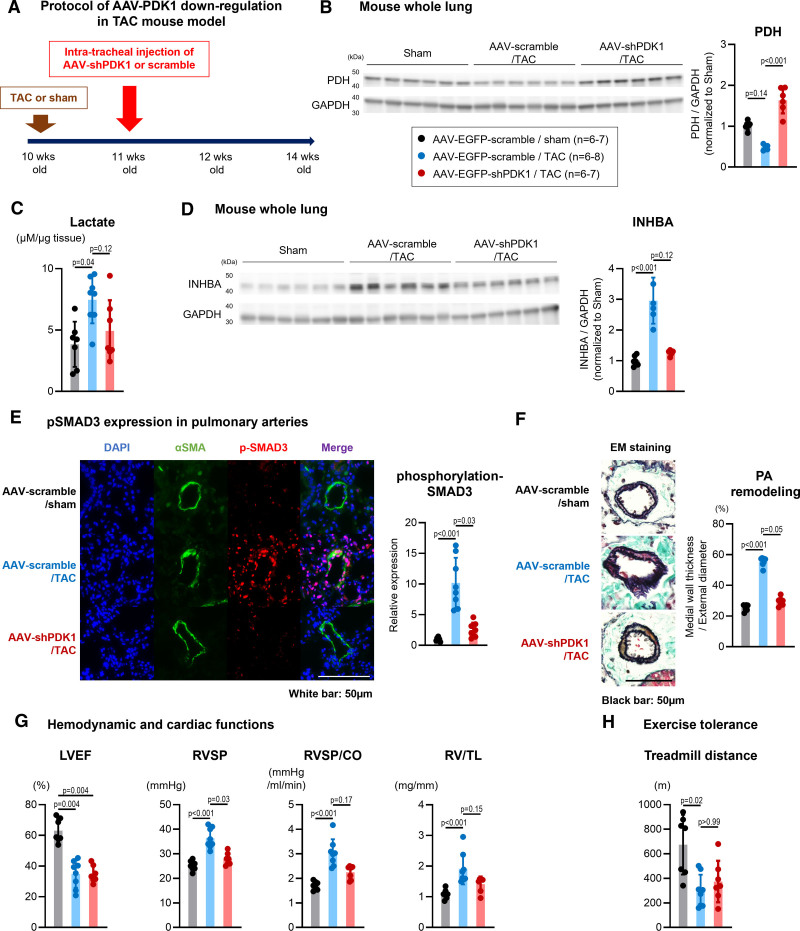
**Inhibition of PDK1 (pyruvate dehydrogenase kinase 1) via adeno-associated virus (AAV) transduction attenuates lactate accumulation, pulmonary artery (PA) remodeling, and cell proliferation signaling in transverse aortic constriction (TAC) mice. A**, Experimental timeline for the inhibition of PDK1 signaling through AAV transduction via intratracheal instillation in TAC mice. **B**, Western blots and quantification of PDH (pyruvate dehydrogenase) and GAPDH expression in lung tissue collected from TAC mice treated with AAV (each n=6). **C**, Lactate levels in the whole lungs of TAC mice treated with AAV. **D**, Western blots and quantification of INHBA (inhibin subunit beta A) and GAPDH expression in the lung tissue collected from TAC mice treated with AAV (n=6). **E**, Representative immunofluorescence images and quantification of αSMA (smooth muscle actin alpha; white), p-SMAD3 (phosphorylated SMAD; purple), and DAPI (4',6-diamidino-2-phenylindole; blue) expression in distal PAs of TAC mice. Scale bar=50 µm. **F**, Representative Elastica Masson (EM) images and quantification of PA remodeling in TAC mice treated with AAV. **G**, Echocardiographic measurements assessing left ventricular ejection fraction (LVEF), right ventricular systolic pressure (RVSP), RVSP/cardiac output (CO) ratio (total pulmonary resistance), and the ratio of right ventricular weight to tibia length (RV/TL) in TAC mice treated with AAV (n=7–8). **H**, Treadmill test performance of TAC mice treated with AAV (n=7–8). Data are presented as mean±SD. Comparisons between each group were analyzed using the Kruskal-Wallis test followed by the Dunn test. AAV-shPDK1 indicates adeno-associated virus-short hairpin INHBA; and EGFP, enhanced green fluorescent protein.

Based on the in vitro data, upregulation of PDK1 alters glucose metabolism and promotes an acidic microenvironment. To assess the role of the PDK1–PDH axis in PAs of TAC mice, we administered AAV6-shPDK1 (short hairpin PDK1) to TAC mice (Figure 6A; Figure S10). AAV-mediated knockdown of PDK1 restored PDH expression and reduced TAC-induced lactate accumulation in the lungs (Figure 6B and 6C). Correspondingly, the upregulation of INHBA and SMAD3 phosphorylation was also attenuated (Figure 6D and 6E). Histological analysis demonstrated reduced PA remodeling in the AAV6-shPDK1 group (Figure 6F). Although echocardiography showed no significant difference in EF between mice treated with AAV6-scramble and AAV6-shPDK1, hemodynamic assessments revealed improvements in RVSP, total pulmonary resistance, and right ventricular weight to tibia length ratios (Figure 6G), along with an increased pulmonary acceleration time (Figure S11). In addition, treadmill testing showed improved exercise tolerance in the AAV6-shPDK1–treated group (Figure 6H). These results suggest that PDK1-mediated metabolic dysfunction contributes to lactate accumulation, which may induce an acidic environment that promotes PA remodeling and hypertension.

## Discussion

The key findings of this study from both human and basic analyses are as follows: (1) RNA sequencing and metabolomics identified INHBA as a key regulator of PA remodeling in group 2 PH, (2) mechanical stretch stress induces acidosis via metabolic dysfunction in PAH PASMCs, (3) acidity elevates INHBA expression in PAs, (4) downregulation of INHBA in the lungs of the group 2 PH model attenuated PA remodeling.

### Shared Pathophysiological Features of PA Remodeling in Group 1 and 2 PH

This study aims to contribute to research on group 2 PH, an area where experimental investigation remains challenging due to limited tissue availability and substantial clinical heterogeneity. In this context, mechanistic studies benefit from being guided by clearly defined objectives and specific phenotypes. Importantly, we do not regard group 1 PH and group 2 PH as having equivalent overall disease profiles. Rather, we drew on established observations that both conditions exhibit certain aspects of PA remodeling. These shared features were used as a reference point to help identify potential pathways that may be relevant to group 2 PH.

Assad et al^13^ identified common genetic signatures between group 1 and 2 PH, particularly those associated with cytoskeletal organization and actin binding, which contribute to smooth muscle cell proliferation, fibrosis, and vascular wall thickening. In addition, mitochondrial abnormalities, including those involving SMCR7, a modulator of dynamin-related protein 1, which regulates mitochondrial fission, have been implicated in both conditions, thereby, reinforcing the hypothesis of overlapping pathophysiological pathways.^13^ These findings highlight the potential for future studies into shared genetic and molecular mechanisms to inform new therapeutic strategies, particularly for CpcPH associated with poor outcomes.

The lack of lung samples and cultured cells from patients with group 2 PH has limited our ability to fully explore the pathogenesis of this condition. To address this issue, we utilized the GEO data from the PAs of group 2 PH. Furthermore, by focusing on PA dilation induced by pressure shifts from the left side in the pathophysiology of group 2 PH, we evaluated the mechanical stretch-induced alterations in PASMCs from both the PAH and control groups. In the combined RNA sequencing–metabolomic analyses using these data, we identified central mechanisms related to cell proliferation that warrant further attention, demonstrating that TGF-β signaling and mitochondrial dysfunction are common promoters in both groups (Figures 1 and 3).

To date, investigations into the pathophysiological mechanisms of group 2 PH have frequently referenced molecular pathways originally identified in group 1 PH, reflecting the limited availability of disease-specific experimental models.^7,34^ These pathways include BMPR2 (bone morphogenetic protein receptor 2),^35,36^ KCNK3 (potassium channel subfamily K member 3),^37,38^ IL (interleukin)-6/STAT3 (signal transducers and activator of transcription 3) signaling,^39,40^ endothelin,^41,42^ 5-HTT (serotonin transporter),^43,44^ eNOS (endothelial nitric oxide synthase),^45,46^ ID proteins (inhibitor of DNA-binding/differentiation proteins),^47,48^ and Hippo signaling.^12,49^ Within this context, our findings suggest that INHBA within the TGF-β signaling axis warrants consideration as a pathway associated with pulmonary vascular remodeling in group 2 PH, particularly at the level of PASMC biology.^19,50^ Rather than indicating a shared disease-defining mechanism between group 1 and group 2 PH, these results support the presence of pathway-level overlap in PASMC signaling. This interpretation is consistent with prior reports of partial molecular convergence and should be regarded as hypothesis-generating, pending validation in group 2 PH–specific experimental systems.

The value of this approach lies in combining contemporary analytical methods—including single-cell data sets and IPA analyses utilizing actual PA of patients with group 2 PH—with the growing availability of open-source patient data. Together, these resources enabled us to propose candidate mechanisms and potential therapeutic targets for group 2 PH, while acknowledging the need for further validation in disease-specific models.

### Stretch-Induced Metabolic Dysfunction and Its Role in PA Remodeling

In this study, we highlighted the stretch-induced accumulation of lactate in PAH PASMCs, indicating a compromised metabolic reserve capacity in accommodating pulmonary vascular dilation. PAH vascular cells are characterized by a metabolic abnormality known as the Warburg effect, wherein glucose metabolism shifts from aerobic (tricarboxylic acid cycle) to anaerobic (lactate accumulation) pathways. This metabolic adaptation supports cell proliferation under anaerobic conditions, such as PH or cancer.^51^ Notably, our findings revealed that stretch stress, which simulates pulmonary dilation caused by pressure overload, exacerbates this effect, specifically in PAH cells.

Importantly, the GEO of the PAs of patients with group 2 PH demonstrated evidence of metabolic dysfunction (Figure 3). A group 2 PH animal model, the mice with TAC, exhibited an increased GPR68 expression in the PAs, indicative of acidosis-related alterations and consistent with these human findings. These results suggest that similar mechanisms may contribute to metabolic dysfunction in the PAs of patients with group 2 PH.

Lactic or acidic alterations have severe consequences, including driving cell type transitions in cancer^52^ and promoting oxidative stress by disrupting cellular metabolism.^53^ Furthermore, previous studies have shown that lactate stimulates cell proliferation in PH.^54,55^

This study elucidated detailed mechanisms by which mechanical stretch promotes c-MYC–PDK1 axis activation and disrupts energy metabolism, leading to lactate accumulation and acidification. Notably, we provide novel in vivo evidence linking enhanced TGF-β signaling and acidic conditions in PAs, predominantly via p38 signaling—both key mediators of cell proliferation. These findings highlight a potential pathogenic mechanism shared across group 1 and group 2 PH.

### Therapeutic Implications and Future Directions

The findings of this study underscore the potential of targeting TGF-β signaling and INHBA as a therapeutic focus for group 2 PH, which is marked by PA remodeling and elevated PVR. Sotatercept, an activin signaling inhibitor, has been approved for PAH treatment and has demonstrated improved clinical outcomes.^21–23^ This drug consists of the Fc domain of human immunoglobulin G fused to the extracellular domain of human activin receptor type IIA, acting as a ligand trap for specific TGF-β superfamily members.^21^ The ongoing phase II CADENCE trial (A Phase 2, Double-Blind, Randomized, Placebo-Controlled Study to Evaluate the Effects of Sotatercept Versus Placebo for the Treatment of Combined Postcapillary and Precapillary Pulmonary Hypertension [Cpc-PH] Due to Heart Failure With Preserved Ejection Fraction [HFpEF]) is evaluating the efficacy and safety of sotatercept for group 2 PH compared with a placebo in patients with CpcPH due to heart failure with preserved ejection fraction. Despite these advancements, limited evidence exists regarding the molecular mechanisms of TGF-β signaling in group 2 PH. In this study, we partially address this gap by providing insight into these mechanisms, which could enhance the understanding of group 2 PH pathogenesis.

Furthermore, we identified a connection between mitochondrial metabolism and INHBA upregulation in PASMCs, presenting a potential therapeutic target for group 2 PH (Graphical Abstract). Phase I trials have explored PDK1 inhibitors in cancer treatment^56^; however, no treatment strategies addressing mitochondrial dysfunction have been developed for group 2 PH. Further research is essential to develop innovative therapeutic approaches targeting TGF-β signaling pathways and mitochondrial metabolism. That could lead to effective strategies for alleviating PA remodeling and improving clinical outcomes in patients with group 2 PH.

### Limitations

This study has several limitations. First, lung tissue and cultured PASMCs from patients with group 2 PH were not available, which restricts the degree to which our findings can be directly connected to disease-specific vascular biology. Second, the plasma concentrations of activin A, follistatin, and FSTL3 may not necessarily reflect PA remodeling, as these proteins are produced by various cell types. Accordingly, the observed increase in activin A should be regarded as an exploratory observation and interpreted with appropriate caution. Third, AAV transduction was not specific to the PASMC layer, and potential effects on endothelial or interstitial cells cannot be excluded, which limits the precision of cell-type–specific interpretation. In addition, while immunofluorescence demonstrated the presence of INHBA expression in endothelial cells, this approach is not well-suited to detect subtle quantitative differences within the thin endothelial layer. Flow cytometry–based analysis did not show a clear difference in endothelial INHBA mRNA expression; however, further detailed studies using refined cell-type–specific approaches will be required to determine whether endothelial INHBA expression is modulated under pathological conditions. Also, the in vivo experiments were conducted exclusively using TAC mice, which, despite being informative, may not fully capture the complexity of group 2 PH pathophysiology in humans. In addition, we primarily focused on molecular and cellular mechanisms without direct confirmation of clinical relevance in human tissues beyond transcriptomics and metabolomics. Furthermore, the use of TAC mice may potentially limit the findings to other forms of PH or different etiologies of group 2 PH. Furthermore, the metabolic dysfunction and mitochondrial changes identified in this study were assessed in isolated experimental systems, which may not completely reflect the in vivo environment. In addition, the potential influence of blood flow–dependent vasodilatory responses on the PA phenotype in TAC mice cannot be excluded.

### Conclusions

In this study, we demonstrated that TGF-β signaling, with INHBA as a key regulator, is common to both group 1 and 2 PH. Stretch-induced lactate production in PASMCs led to acidosis, promoting INHBA expression and cell proliferation. TGF-β signaling, including INHBA, is crucial in the pathogenesis of group 2 PH, highlighting its potential as a therapeutic target.

## ARTICLE INFORMATION

### Acknowledgments

The authors are grateful to the laboratory members in the Department of Cardiovascular Medicine at Tohoku University, especially Yumi Watanabe, Hiromi Yamashita, and Kaori Miyamura, for their valuable technical assistance.

### Sources of Funding

The present study was supported by the Japan Agency for Medical Research and Development (JP22ek0210149 to S. Yasuda and JP22K16126 to T. Satoh).

### Disclosures

None.

### Supplemental Material

Supplemental Methods

Tables S1–S3

Figures S1–S11

Major Resources Table

ARRIVE Guidelines

Western Blots

References 25,26,30,32,57–76

## Supplementary Material


