# Supplementary material for: Pathogenesis of Pulmonary Artery Remodeling: TGF-Beta Signaling and Inhibin Subunit Beta A in Group 1 and 2 Pulmonary Hypertension
Source: Arterioscler Thromb Vasc Biol. 2026 Jan 22;46(3):e322506. doi: 10.1161/ATVBAHA.125.322506 (PMC12931867; doi:10.1161/ATVBAHA.125.322506)
Supplement: Supplementary file 1 [file atv-46-e322506-s001.pdf]

Supplemental materials

Pathogenesis of Pulmonary Artery Remodeling: TGF-beta Signaling and Inhibin Subunit Beta A in Group 1 and 2 Pulmonary Hypertension

Running Title: *Yamada Y, et al.; INHBA in Group2 PH*

Yusuke Yamada[1], Taijyu Satoh[1,2], Nobuhiro Yaoita[1], Kaito Yamada[1], Naoki Chiba[1], Kohei Komaru[1], Kotaro Nochioka[1], Saori Yamamoto[1], Haruka Sato[1], Nobuhiro Kikuchi[1], Takashi Nakata[1], Shinichiro Sunamura[3], Takumi Inoue[1], Hideka Hayashi[1], Hideaki Suzuki[1], Shunsuke Tatebe[1], Hiroyuki Takahama[1], Hisashi Oishi[4], Satoshi Miyata[5], Yoshinori Okada[4], Satoshi Yasuda[1]

Affiliations:

[1] Department of Cardiovascular Medicine, Tohoku University Graduate School of Medicine, Japan

[2] Department of Medical Science and Innovation, SiRIUS Institute of Medical Research, Tohoku University, Japan

[3] Department of Cardiology, Sendai City Medical Center, Sendai Open Hospital, Sendai, Japan.

[4] Department of Thoracic Surgery, Institute of Development, Aging and Cancer, Tohoku University, Japan

[5] Teikyo University Graduate School of Public Health, Japan

Address for Correspondence:

Satoshi Yasuda, MD, PhD

Department of Cardiovascular Medicine, Tohoku University Graduate School of Medicine,

Sendai, 980-8574; E-mail: satoshi.yasuda.c8@tohoku.ac.jp

Supplemental Methods

Supplemental Table S1-3

Supplemental Figures S1-11

Major Resources Table

Preparation of Mouse INHBA and PDK1 Plasmids and Lung- specific INHBA and PDK1 downregulation using adeno associated virus vector

Firstly, mice were subjected to TAC or Sham following described methods in this main manuscript.[26] Subsequently, one week later, to downregulate mouse INHBA or PDK1 expression, mice received a single intratracheal instillation of AAV6-CMV encoding GFP and either shINHBA or shPDK1 (1×10^{11} gc/mouse, Vector Builder Inc., Chicago, USA), along with saline. Then, 3 weeks later, the levels of INHBA or PDH expression in the lung of the mice were quantified using Western blotting. Statistical power of all comparisons with significant difference was calculated based on sample size, minimum effect of interest, variability (SD/means difference), and significant levels (α=0.05) using G★Power 3.1.9.7 as previously described.[57] The appropriate sample size (ie, n=6-8) was determined to achieve statistical power of >80% for all comparisons. The minimum statistical power was 82% for comparing RVSP, which was most important parameter to evaluate pulmonary hypertension in this animal experiments, of three groups of AAV treated experiments of TAC mice, with n=6.

Ultrasonography

Ultrasonography was conducted both at rest and under isoflurane anesthesia using a Vevo 2100 ultrasound system (FUJIFILM Visualsonics Inc., Toronto, Canada). For measurements, LV and RV dimensions and functions were assessed in B-mode, M-mode and pulse wave doppler mode as previously detailed.[30] All assessments were completed within a 5-minute timeframe while the subjects were under isoflurane anesthesia.

Treadmill Exercise Test

To assess exercise capacity, the maximal walking distance was measured on a motor-driven treadmill (MK-680, Muromachi Kikai, Tokyo, Japan). The initial treadmill speed was set at 5 m/min and subsequently increased by 5 m/min every 5 minutes. This progressive increase continued until reaching 20 m/min for mice and rats or until the animals exhibited signs of fatigue, which was determined when the animal accepted the electrical stimulus 3 times within 10 sec, as previously described.[25]

Blood Pressure measurement

Blood pressure was measured at 4 weeks after the TAC procedure in mice, and at 18-week-old in SHR with the tail-cuff system (MK-2000ST NP-NIBP Monitor, Muromachi Kikai) without anesthesia.

Assessment of Right Ventricular Hypertrophy

The whole heart was excised, and the right ventricular wall was carefully separated from the left ventricle and septum. To quantify the extent of right or left ventricular hypertrophy (RVH or LVH), the ratio of weight of the right or left ventricle plus septum to the tibia length (RV/TL or LV+S/TL) was calculated.

Histological Analysis

Following hemodynamic measurements, the heart and lungs were perfused entirely with cold phosphate-buffered saline (PBS) at physiologic pressures. Subsequently, they were fixed in a 10% formaldehyde solution or 4% paraformaldehyde solution (PFA) for a period of 24 hours and embedded in paraffin or OCT (optimal Cutting Temperature), as previously outlined.[58] Sections with a thickness of 3 μm were prepared on slides. To evaluate pulmonary arterial remodeling, sections on the slides underwent staining for Elastica Masson (EM). In each section, a total of 60-80 vessels, specifically those with external diameters falling within the range of 50-100 μm, were examined using a fluorescence microscope (BZ9000 or BZX800, Keyence, Tokyo, Japan).[30] The external diameter and medial wall thickness of pulmonary arteries were quantified using ImageJ (NIH, Bethesda, USA). Measurements were taken from 10 muscular arteries per lung section, with the arterial external diameter ranging from 50-100 μm, across 6-8 mice or rats per group. The medial index (%) was calculated as follows: (mean medial wall thickness/mean external diameter) × 100, following established procedures.[26]

Immunofluorescence Staining

For immunofluorescence staining, mouse or rat lung sections on slides embedded in OCT were incubated overnight and stained using the following antibodies: α-smooth muscle actin (1:100, αSMA-cy3, C6198, Sigma-

Aldrich, St. Louis, USA, or αSMA Alexa Fluor® 488 Conjugate, #46469, Cell Signaling Technology (CST), Danvers, USA), Activin A (1:40, AF338, R&D Systems, Boston, USA), CD31 (1:100, ab28364, Abcam, Cambridge, UK), phosphorylation-SMAD3 (1:100, ab52903, Abcam), GPR68 (1:250, #720277, Thermo Fisher Scientific, Waltham, USA), and DAPI (VECTASHILED Mounting Medium, H-1500-10, Vector Laboratories, Newark, USA), followed by corresponding secondary fluorescently labeled antibodies (Life Technologies, Carlsbad, USA). In each section, a total of 60–80 vessels, specifically those with external diameters falling within the range of 50-100 µm, were meticulously examined using a fluorescence microscope (BZ9000 or BZX800, Keyence). This analysis was conducted to assess expression of each target protein in lung sections.

Lactate assay in the lungs of mice

Lactate concentrations were measured by a colorimetric assay according to the manufacturer's instructions (MAK570, Sigma-Aldrich), with homogenates of mouse lung tissue.

Mechanical Stretch Stress to cultured PASMC or PAECs

To examine the cellular effects of mechanical stress, human pulmonary arterial smooth muscle cells (PASMCs) or pulmonary arterial endothelial cells (PAECs) were initially seeded in 10cc stretch chamber (Strex, Osaka, Japan) using Dulbecco's Modified Eagle Medium (DMEM) (Thermo Fisher Scientific) containing 10% fetal bovine serum (FBS) (Cytiva, Tokyo, Japan) or endothelial cell basal medium containing endothelial growth supplement (EGM) (Promo Cell, Heidelberg, Germany). These cells were subjected to cyclic stretch with 10% elongation at 1 Hz for up to 24 or 48 hours using Strex system (Strex).[26] After the stimulus, total cell lysates were collected. Briefly, PASMCs or PAECs were washed with cold PBS or Tris-buffered saline (TBS) and harvested on ice in cell lysis buffer (#9803, CST, Danvers, USA) with protease inhibitor cocktail (P8340, Sigma-Aldrich) and phosphatase inhibitor cocktail (#5870, CST). Changes in intracellular pH were measured with Intracellular pH (pHi) Detection kit (P35372, Thermo Fisher Scientific) in accordance with the manufacturer's protocol, using SpectraMax i3X (Molecular devices, California, USA).

Transfection with siRNA

To downregulate INHBA or c-MYC expression, human PASMCs were transfected with siRNA specifically targeting INHBA (S7434, Thermo Fisher Scientific), c-MYC (VHS40785, Thermo Fisher Scientific) or with a scramble siRNA, which served as a negative control. The transfection was carried out using 10 nmol/L of siRNA with Lipofectamine RNAiMAX Reagent (Thermo Fisher Scientific) in Opti-MEM reduced serum medium (Thermo Fisher Scientific), following the manufacturer's instructions. Following transfection, cells were cultured in a serum-containing medium for 24 hours to facilitate the downregulation of INHBA or c-MYC protein, followed by stretch stress.

Cell proliferation analysis

Cell proliferation was determined by 5-bromo-2'-deoxyuridine (BrdU) incorporation. PASMCs were subjected to cyclic stretch for 24 hours. 10 μM BrdU was added to the medium two hours before the end of the stretch period. Then the medium was removed, and cells were washed twice with cold PBS. After cells were harvested by trypsinization (TrypLE Select Enzyme, Thermo Fisher Scientific) and counted (Countess 3FL Automated Cell Counter, Thermo Fisher Scientific), they were seeded into 96-well cell culture plates at a density of 1×10^4 cells /well. BrdU incorporation assay was performed using a cell proliferation ELISA (#11647229001, Roche Diagnostics GmbH, Mannheim, Germany) according to the manufacturer's instructions.

Western Blotting Analysis

Human PASMCs or PAECs were initially seeded in 100-mm dishes using DMEM containing 10% FBS or EGM. These cells were allowed to adhere for 24 hours, after which they were washed twice and subjected to serum-starvation in a serum-free medium for an additional 24 hours. Following these, the PASMCs were treated with recombinant Activin A protein (100 ng/ml, 11348-AC, R&D Systems) or a vehicle for 6 or 24 hours, one hour after the treatment of the JNK inhibitor (20μM, SP600125, P3160, Tokyo Chemical Industry, Tokyo, Japan), ERK inhibitor (20μM, PD98059, #9900, CST), p-p38 inhibitor (10μM, SB203580, F0864, Tokyo Chemical Industry) or a vehicle. In some experiments, PASMCs were incubated with DMEM adjusted to pH

8.5, 7.5, 6.5, or 5.5 for 24 hours. After the respective incubation period, the cells were washed with cold PBS or TBS and then lysed using cell lysis buffer supplemented with protease inhibitor cocktail and phosphatase inhibitor cocktail. Lung homogenates or cell lysates from human PASMCs and PAECs were centrifuged at 15,000 g for 20 minutes at 4 °C, and the supernatants were collected. Protein concentrations were normalized using the BCA Protein Assay Kit (23225, Thermo Fisher Scientific). Equal volumes of the supernatants were loaded into each well for sodium dodecyl sulfate-polyacrylamide gel electrophoresis gel (SDS-PAGE, Bio-Rad, Hercules, USA) and subsequently transferred to polyvinylidene difluoride membranes (PVDF, Cytiva). After transfer, the membranes were blocked for 1 hour at room temperature using a 5% bovine serum albumin (BSA, A7030, Sigma-Aldrich)) solution in TBS with Tween 20 (TBS-T). The primary antibodies used were as follows: INHBA (1:1000, ab128958, Abcam), p-JNK (1:1000, #4668, CST), JNK (1:1000, #9252, CST), p-ERK1/2 (1: 1000, #9101, CST), ERK1/2 (1: 1000, #9102, CST), p-SMAD3 (1:2000, ab52903, Abcam), SMAD2/3 (1:1000, #8685, CST), p-p38 (1:1000, #9211, CST), p38 (1:1000, #9212, CST), c-MYC (1:1000, #9402, CST), PDK1 (1:1000, #3062, CST), PDH (1:1000, #2784, CST), VE-cadherin (1:1000, ab33168, Abcam), ACTA2 (1:1000, ab5694, Abcam), PECAM1 (1:1000, 11265-1-AP, Proteintech, Tokyo, Japan) and GAPDH (1:2000, #2118, CST), followed by secondary antibodies (Anti-rabbit IgG HRP-linked Antibody, 1:5000, #7074, CST). The protein bands were visualized using the enhanced chemiluminescence system (ECL Prime Western Blotting Detection Reagent, Cytiva). Densitometric analysis was carried out using ImageJ software.

RNA Isolation and Real-time PCR

Total RNA isolation from human PASMCs and PAECs were carried out using the RNeasy Plus Mini Kit (Qiagen K.K., Tokyo, Japan) in accordance with the manufacturer's protocol. The extracted total RNA was then converted into complementary DNA (cDNA) using the PrimeScript RT Master Mix (Takara Bio, Kusatsu, Japan). Primer sets for the following genes were employed: human GAPDH (Primer Set ID: HA067812), INHBA (Primer Set ID:/HA296969), SEPRINE1 (Primer Set ID: HA193370), CCND1 (Primer Set ID: HA255652), and CCN2 (Primer Set ID: HA338261), CDH5 (Primer Set ID: HA352558), PECAM1 (Primer Set

ID: HA275563), S100A4 (Primer Set ID: HA378180), ACTA2 (Primer Set ID: HA133460), TAGLN (Primer Set ID: HA301354), SNAI1 (Primer Set ID: HA328103), SNAI2 (Primer Set ID: HA357045), mouse GAPDH (Primer Set ID: MA050371), and INHBA (Primer Set ID: MA179598), all of which were acquired from Takara Bio. The primer sequences are listed in **Table S3**. Following reverse transcription, quantitative real-time PCR was conducted using the CFX 96 Real-Time PCR Detection System (Bio-Rad) with either TB Green Premix Ex Taq II (Takara Bio) for TB Green probes. The Ct (cycle threshold) value for all samples was determined using CFX Manager Software (version 2.0, Bio-Rad), normalized to the housekeeping gene GAPDH, and the relative fold change was calculated using the ΔΔCt method.[26]

RNA isolation and RNA sequence

RNA was isolated from low-passage (4–7) cells using the RNeasy kit with on-column DNA digestion. The RNA sequencing was conducted by Rhelixa (Tokyo, Japan). The quality of the RNA was assessed using the Nanodrop (Thermo Fisher Scientific) with all samples exhibiting an RNA integrity number greater than 6. To prepare Poly(A) RNA, the Poly(A) mRNA Magnetic Isolation Module (New England Biolabs, Ipswich, USA) was used. For RNA sequencing, libraries were prepared using the NEBNext® Ultra™II Directional RNA Library Prep Kit for Illumina® (New England Biolabs). Sequencing was performed on a NovaSeq 6000 system (Illumina Inc., San Diego, USA). On average, 30 million single reads, each 50 base pairs in length, were generated for each sample.

RNA sequence analyses

All analyses were conducted by Rhelixa following this procedure. Prior to analysis, the quality of the raw paired-end sequence reads was assessed using FastQC.[59] Subsequently, sequences with low quality (below a Phred score of 20) and adapter sequences were removed using Trimmomatic software (Version 0.38) .[60] The trimmed reads were then aligned to the human reference genome hg38 using the RNA sequence aligner HISAT2 (Version 2.2.0).[61] The resulting HISAT2 alignment files in .sam format were further converted into .bam files using Samtools (Version 1.9) .[62] To estimate the abundance of uniquely mapped reads, the .bam

files were subjected to analysis with featureCounts (Version 1.6.3) .[63] Raw read counts were subsequently normalized to transcripts per million (TPM). Differential expression analysis was performed using the DESeq2 R/Bioconductor package[64]. Differentially expressed genes (DEGs) were identified using the following criteria: |log2FC (Fold Change)| greater than 1 and an adjusted p-value less than 0.05, applying the Benjamini and Hochberg (BH) method for multiple testing correction.

The RNA sequencing count data for PAs of patients with Group2 PH were obtained from the NCBI Gene Expression Omnibus (GEO) database (GSE236251). The data were analyzed using iDEP (integrated Differential Expression and Pathway analysis).[65] For preprocessing, the default settings in iDEP were applied, including a minimum CPM (counts per million) threshold of 0.5, requiring at least 1 library to express the gene. The count data were then normalized using log2-transformed after adding a pseudo-count of 4, as implemented in the edgeR method within the iDEP.[66] The transformed data were used for clustering and PCA (Principal Component Analysis).

Subsequent differential expression analyses were conducted to compare pulmonary hypertension with left heart disease (PH-LHD) samples to two distinct groups: Donor samples and LHD without PH samples. Differential expression testing was performed using DESeq2 via the iDEP,[64] and results were used to identify significantly regulated genes between the groups.

scRNA sequence data analysis

This study utilized isolated pulmonary artery of human. Single-cell RNA sequencing (scRNA-seq) dataset of PH patients (n=3) and control pulmonary artery from donor (n=3) was obtained from the GEO database (GSE228644).[32] Initial data processing was conducted using the Seurat package (version 5.0.1) in the R environment (version 4.3.2). The SCTransform function was applied to the dataset for normalization and variance stabilization. Following normalization, PCA was performed using the RunPCA function in Seurat.[67] Subsequently, uniform manifold approximation and projection (UMAP) were employed for dimensionality reduction and visualization, using the RunUMAP function with the top 30 principal components (PCs) and a resolution of 0.1. The dataset was further processed for imputation of missing values, a common issue in scRNA-seq data due to

dropout events. Adaptive Low-Rank Approximation (ALRA) was applied using the RunALRA function in SeuratWrappers to impute missing values, which are common in single-cell RNA-seq data due to dropout events.[68] Next, six single-cell datasets were integrated using Canonical Correlation Analysis (CCA), implemented via the IntegrateLayers() function with method = CCAIntegration in the Seurat package.[69] After integration, cell annotation was performed using both an automatic annotation tool and manual annotation. For the manual annotation, specific markers were utilized to identify cell types: ACTA2 for smooth muscle cells, CDH5 for endothelial cells, and DCN for fibroblasts (**Figure 1B**).[32,70]

Feature plots were generated for imputed alra datasets using the FeaturePlot function. We selected key features such as INHBA, PCNA and CCND1 for visualization (**Figure 1B**).

To investigate the relationship between the expression levels of the genes in smooth muscle cluster, a scatter plot analysis was performed. The FeatureScatter function from the Seurat package was utilized to create a scatter plot (**Figure S1**). To quantitatively assess the correlation between INHBA, PCNA and CCND1 expression levels in smooth muscle cluster, the Pearson correlation coefficient was calculated. This was achieved using the stat_cor function from the ggpubr (version 0.6.0) package, which was added to the scatter plot.

Metabolome analyses

Metabolome analysis was conducted according to HMT's ω Scan package (Human Metabolome Technologies (HMT), Tsuruoka, Japan), using capillary electrophoresis Fourier transform mass spectrometry (CE-FTMS) based on the methods described previously.[71] Briefly, CE-FTMS analysis was carried out using an Agilent CE capillary electrophoresis system equipped with a Q Exactive Plus (Thermo Fisher Scientific). The systems were controlled by Agilent MassHunter software (version 2.19.0.2) and connected by a fused silica capillary (50μm i.d.×80cm total length) with commercial electrophoresis buffer (H3301-1001 for cation and anion analyses, respectively, HMT) as the electrolyte. The spectrometer was scanned from m/z 50 to 1,000 in positive mode, and from m/z 70 to 1,050 in negative mode, respectively.[71] Peaks were extracted using MasterHands, automatic integration software (HMT) in order to obtain peak information including m/z, peak area, and migration time (MT).[72] Signal peaks corresponding to isotopomers, adduct ions, and other product ions of known metabolites

were excluded, and the remaining peaks were annotated according to HMT's metabolite database based on their m/z values and MTs. Areas of the annotated peaks were then normalized to internal standards and sample volume in order to obtain relative levels of each metabolite. Detected metabolites were plotted on metabolic pathway maps using VANTED software.[73]

For additional experiments of cultured PASMCs stretched for 24 hours, Pyruvate (MAK071, Sigma-Aldrich), Acetyl-Coenzyme A (COA) (MAK039, Sigma-Aldrich), Lactate (MAK064, Sigma-Aldrich) were evaluated by commercially available kits in accordance with the manufacturer's protocol.

Endothelial-to-Mesenchymal Transition (EndMT) evaluation

Based on previous reports[74], To evaluate the EndMT, human PAECs were incubated with Activin A (100ng/ml) for 1 to 7 days and evaluated by western blotting and real-time PCR using EndMT makers (S100A4, ACTA2, TAGLN, Snail1, and Snail2), endothelial markers (CDH5 and PECAM1).

For immunofluorescence staining, Human PAECs were seeded in glass-bottom dishes at a density of 15,000 cells per well in EGM. The following day, PAECs were stimulated with Activin A (100ng/ml) for 1 to 7 days. Subsequently, the cells were fixed with PFA. Immunostaining was performed using antibodies specific to VE-cadherin (1:400, ab33168, Abcam) and αSMA (1:200, ab5228, Abcam). The stained slides were examined using a fluorescence microscope (BZ9000, Keyence).

Crystal violet staining

Crystal violet solution (V5265, Sigma-Aldrich) was added to cover the bottom of the plate of cultured PASMCs which were fixed with PFA, and the plate was put on a shaker for 30 min. The plate was rinsed three times with PBS to remove crystal violet and allowed to dry overnight. The stained cells were imaged under a fluorescence microscope (BZ9000, Keyence) with a 4× objective in 5 unique fields of view per plate. The images were quantified using a custom ImageJ software.[75,76]

Lung cell isolation and Flow Cytometry (FCM)

The lungs were rinsed with Hanks' balanced salt solution (14175-095, Thermo Fisher Scientific) containing 10 mM 4-(2-hydroxyethyl)-1-piperazineethanesulfonic acid (HEPES) (25-060-CI, Corning Incorporated, Corning, USA) and 1% of BSA. The lungs were then minced with fine-tip scissors. Subsequently, the lungs were digested with collagenase Type IV (0.5%, 17104019, Thermo Fisher Scientific), Dispasea II (2.4 U/ml, 17105041, Thermo Fisher Scientific) and DNase I (50 µg /ml, 10104159001, Roche) at 37 °C for 45 minutes. The lung suspensions were filtered through 35 µm cell strainers with 5 mL Tube (Corning) and centrifuged at 400 g at 4 °C for 5 minutes. The cell pellets were resuspended in stain buffer containing BSA (554657, Becton, Dickinson and Company (BD), Franklin Lakes, USA). After the lung cells were isolated, the total number of cells was counted.

The surface antigens of the digested lung cells were stained with PE-conjugated anti-Mouse CD31 (1:100, 561073, BD), APC-conjugated anti-Mouse CD45 (1:100, 559864, BD) and APC-conjugated anti-Mouse CD326 (1:100, 563478, BD) for 45 minutes in the dark at 4 °C. SYTOX Blue (1 µM, S34857, Thermo Fisher Scientific) was used to evaluate cell viability. Antibody specificity was assessed using unstained controls.

Mouse PAECs were defined as $CD45^{-}/CD326^{-}/CD31^{+}$ cells, which were analyzed and sorted using FACSAria II cell sorter (BD). FACSDiva software (BD) was used to collect and visualize data for all flow cytometry studies. Total RNA was isolated from mouse PAECs using the RNeasy Plus Micro Kit (Qiagen K.K.) according to the manufacturer's protocol. Subsequently, as described above, the extracted total RNA was converted to cDNA and real-time PCR was performed.

ELISA

Following collection, the plasma samples were promptly subjected to centrifugation for 15 minutes at 1000 g and aliquots were then preserved at -80°C. Plasma levels of the target factors were quantified using commercially available ELISA kits according to the manufacturer's instructions, specifically for activin A (DAC00B, R&D Systems), follistatin (DFN00, R&D Systems) and FLRG (DFLRG0, R&D Systems). Each experiment was conducted in duplicate to ensure data accuracy and consistency.

Supplemental Table S1. Characteristics of patients with Group 2 PH providing plasma

		Total	PVR ≤2 WU	PVR >2 WU	P value
Number		81	37	44	
Age (years)		68 (58-77)	67 (55-77)	71 (59-78)	0.3333
male (n, %)		48 (59%)	30 (81%)	18 (41%)	**0.0002**
BMI (kg/m^2)		23.6 (20.2-26.1)	23.7 (21.7-25.5)	23.5 (19-27.0)	0.6321
Atrial fibrillation (n, %)		21 (26%)	6 (16%)	15 (34%)	0.0797
Laboratories					
Hb (g/dl)		12.9 (11.2-14.3)	13.7 (11.5-14.9)	12 (11.0-14.2)	0.0530
T-bil (mg/dl)		0.5 (0.7-1.1)	0.8 (0.7-1.2)	0.8 (0.6-1.2)	0.8974
eGFR (ml/min/1.73m2)		52 (36-75)	55 (37-77)	50 (36-71)	0.5129
UA (mg/dl)		5.6 (4.8-7.4)	5.6 (4.9-7.8)	5.6 (4.6-6.9)	0.2754
BNP (pg/ml)		208 (92-403)	229 (88-470)	205 (92-356)	0.8811
LDL-choresterol (mg/dl)		88 (70-109)	101 (72-111)	84 (67-107)	0.2359
HbA1c (%)		5.9 (5.6-6.3)	5.9 (5.6-6.2)	6.1 (5.6-6.6)	0.4616
Hemodynamics					
HR (bpm)		69 (61-80)	73 (60-84)	68 (61-78)	0.2122
RAP (mmHg)		7 (4-11)	7 (4-10)	7 (4-13)	0.5748
PAP (mmHg)					
	mean	27 (23-32)	25 (22-29)	29 (24-35)	**0.0049**
	systolic	43 (36-50)	38 (32-44)	45 (38-58)	**0.0009**
	diastolic	18 (15-22)	18 (13-21)	18 (15-25)	0.1550
PAWP (mmHg)		19 (16-22)	19 (17-22)	19 (16-23)	0.7177
CO (L/min)		3.7 (2.9-4.4)	3.9 (3.4-5.5)	3.3 (2.7-4.1)	**0.0015**
CI (L/min/min2)		2.18 (1.87-2.64)	2.37 (1.90-2.88)	2.1 (1.76-2.43)	**0.0249**
PVR (Woods Units)		2.06 (1.87-2.64)	1.38 (1.01-1.56)	3.1 (2.34-4.26)	**<0.0001**
AoP (mmHg)					
	mean	120 (105-133)	119 (103-132)	120 (103-144)	0.3960
	systolic	69 (58-76)	70 (58-74)	69 (57-78)	0.9621
	diastolic	86 (77-95)	85 (74-96)	86 (78-96)	0.6764
SVR (Woods Units)		21.2 (16.4-27.5)	17.9 (14.7-23.9)	22.5 (18.1-32.2)	**0.0068**
SaO2 (%)		95.2 (94-96.8)	96 (94.6-97.0)	95 (92.9-96.5)	0.0597
SvO2 (%)		63.4 (58.5-66.2)	65 (60.2-67.8)	62.5 (54.6-65.8)	0.0405
Echocardiography					
LVDd (mm)		54 (47-60)	59 (50-66)	51 (46-55)	**0.0030**
LVDs (mm)		38 (33-51)	46 (36-59)	36 (31-44)	**0.0021**
IVS (mm)		9 (8-10)	9 (8-11)	10 (7-11)	0.8108
LVEF (%)		48 (32-62)	39 (24-57)	56 (36-67)	**0.0130**
LA (mm)		46 (41-52)	46 (40-53)	46 (42-52)	0.9137

E/e'	13.8 (11.5-23)	12.3 (9.9-16.8)	17.6 (12.0-24.2)	**0.0105**
RA (mm)	43 (37-49)	41 (37-46)	44 (37-50)	0.5073
RVD (mm)	33 (29-38)	33 (29-38)	33 (30-39)	0.5189
RVFAC (mm)	35 (26-39)	35 (26-38)	35 (25-40)	0.8182
TAPSE (mm)	16 (13.3-18.7)	15.9 (12.7-18.0)	16.4 (13.4-19.3)	0.5665
TR-PG (mmHg)	33 (24-40)	26 (20-36)	36 (31-43)	**0.0003**
Pulmonary function				
TLC (% predcited)	105 (102-110)	105 (104-111)	101 (68-124)	0.6625
FVC (% predcited)	92 (77-101)	95 (77-105)	991 (75-101)	0.3988
FEV_1 (% predcited)	83 (66-97)	88 (64-98)	82 (58-98)	0.7785
FEV1/FVC (%)	74 (70-79)	74 (72-78)	75 (69-81)	0.5821
DLCO (% predcited)	76 (68-101)	91 (77-110)	75 (61-107)	0.3951
DLCO/VA (% predcited)	99 (89-108)	93 (87-109)	106 (60-112)	1.0000
Medications (n, %)				
ACEi/ARB	30 (37%)	12 (32.4%)	18 (40.9%)	0.493
ARNI	21 (25.5%)	12 (32.4%)	9 (20.5%)	0.3091
MRA	47 (58%)	22 (59.5%)	25 (56.8%)	0.8254
beta blocker	57 (70.4%)	24 (64.9%)	33 (75%)	0.3403
SGLT2i	41 (50.6%)	17 (46%)	24 (54.6%)	0.5067
Loop diuretic	51 (63%)	20 (54.1%)	31 (70.5%)	0.1671

Continuous variables are expressed as median and interquartile range, and categorical variables as numbers and percentages. Comparisons of parameters were performed with the Mann–Whitney U test for continuous variables or the Fisher's exact test for categorical variables.
ACEi, angiotensin-converting enzyme inhibitor; AoP, aortic pressure; ARB, angiotensin receptor blocker; ARNI, angiotensin receptor neprilysin inhibitor; BMI, body mass index; BNP, brain natriuretic peptide; CI, cardiac index; DLCO, diffusing capacity of the lungs for carbon monoxide; DLCO/VA, DLCO divided by alveolar volume; e', peak early diastolic mitral annular velocity; E, early diastolic filling velocity; eGFR, estimated glomerular filtration rate; FEV1, forced expiratory volume in one second; FVC, forced vital capacity; Hb, haemoglobin; HbA1c, glycated haemoglobin; HR, heart rate; LA, left atrial diameter; LDL, low density lipoprotein; LVEF, left ventricular ejection fraction; LVDd, left ventricular end-diastolic diameter; MRA, mineralocorticoid receptor antagonist; PAP, pulmonary arterial pressure; PAWP, pulmonary arterial wedge pressure; PVR, pulmonary vascular resistance; RA, right atrial diameter; RAP, right atrial pressure; RVDd, right ventricular diastolic diameter; RVFAC, right ventricular fractional area change; SaO_2, arterial oxygen saturation; SGLT2i, sodium–glucose co-transporter 2 inhibitor; SvO_2, mixed venous oxygen saturation; SVR, systemic vascular resistance; TAPSE, tricuspid annular plane systolic excursion; T-bil, Total bilirubin; TLC, total lung capacity; TR-PG, trans-tricuspid pressure gradient; UA, uric acid.

Supplemental Table S2. Characteristics of patients with PAH providing cultured PASMCs.

**Sex**	**Age**	**Diagnosis**	**mPAP (mmHg)**	**Description**
Male	31	PAH associated CHD	44	Bilateral lung transplantation
Female	36	Idiopathic PAH	67	Bilateral lung transplantation
Female	42	Idiopathic PAH	35	Bilateral lung transplantation
Female	39	Idiopathic PAH	73	Bilateral lung transplantation
Female	29	Idiopathic PAH	96	Bilateral lung transplantation
Male	40	PAH associated CHD	27	Bilateral lung transplantation
Male	33	Idiopathic PAH	57	Bilateral lung transplantation
Male	25	Idiopathic PAH	83	Bilateral lung transplantation

PAH: pulmonary arterial hypertension, CHD: congenital heart disease

Supplemental Table S3. Primer sequences for Real-time PCR

**Human gene**		**Primer sequence**
GAPDH	Forward	GCACCGTCAAGGCTGAGAAC
	Reverse	TGGTGAAGACGCCAGTGGA
INHBA	Forward	AGAAGCTGTTGTATGGGTCAGAGA
	Reverse	GCTGAGACATGTGGTTTGCTTC
SERPINE1	Forward	CTGCATGACCTACCAGGACAGAAC
	Reverse	CAGCCGGAAATGACACATTGA
CCND1	Forward	TCCTGGATGTTGTGTGTATCGAGAG
	Reverse	ACTTGCGCGTCACAGGACAG
CCN2	Forward	TGTGCATGGTCAGGCCTTG
	Reverse	CTCGGTATGTCTTCATGCTGGTG
CDH5	Forward	CAGACCGCCGTCTAACTCAAAG
	Reverse	TCCAGGCAGATAGGCACCAG
PECAM1	Forward	CCTCCAGCCCTAGAAGCCAATTA
	Reverse	CTCAAAGACTGAGTCAGGCCAGTG
S100A4	Forward	GCCCTGGATGTGATGGTGTC
	Reverse	CTTGGAAGTCCACCTCGTTGTC
ACTA2	Forward	ATTGCCGACCGAATGCAGA
	Reverse	ATGGAGCCACCGATCCAGAC
TAGLN	Forward	CCTCTGACACATGCGGCTTTAC
	Reverse	GAGTCATTCCAGGTCGGCATC
SNAI1	Forward	CCAGTGCCTCGACCACTATG
	Reverse	TTAGAGTCCTGCAGCTCGCTGTA
SNAI2	Forward	CCTGGTTGCTTCAAGGACACATTA
	Reverse	GCAGATGAGCCCTCAGATTTGAC
**Mouse gene**		**Primer sequence**
GAPDH	Forward	TGTGTCCGTCGTGGATCTGA
	Reverse	TTGCTGTTGAAGTCGCAGGAG
INHBA	Forward	TTCCAGCGGATATTCTGATGTTTG
	Reverse	GGGCATTGCTGAATACTGCCTA

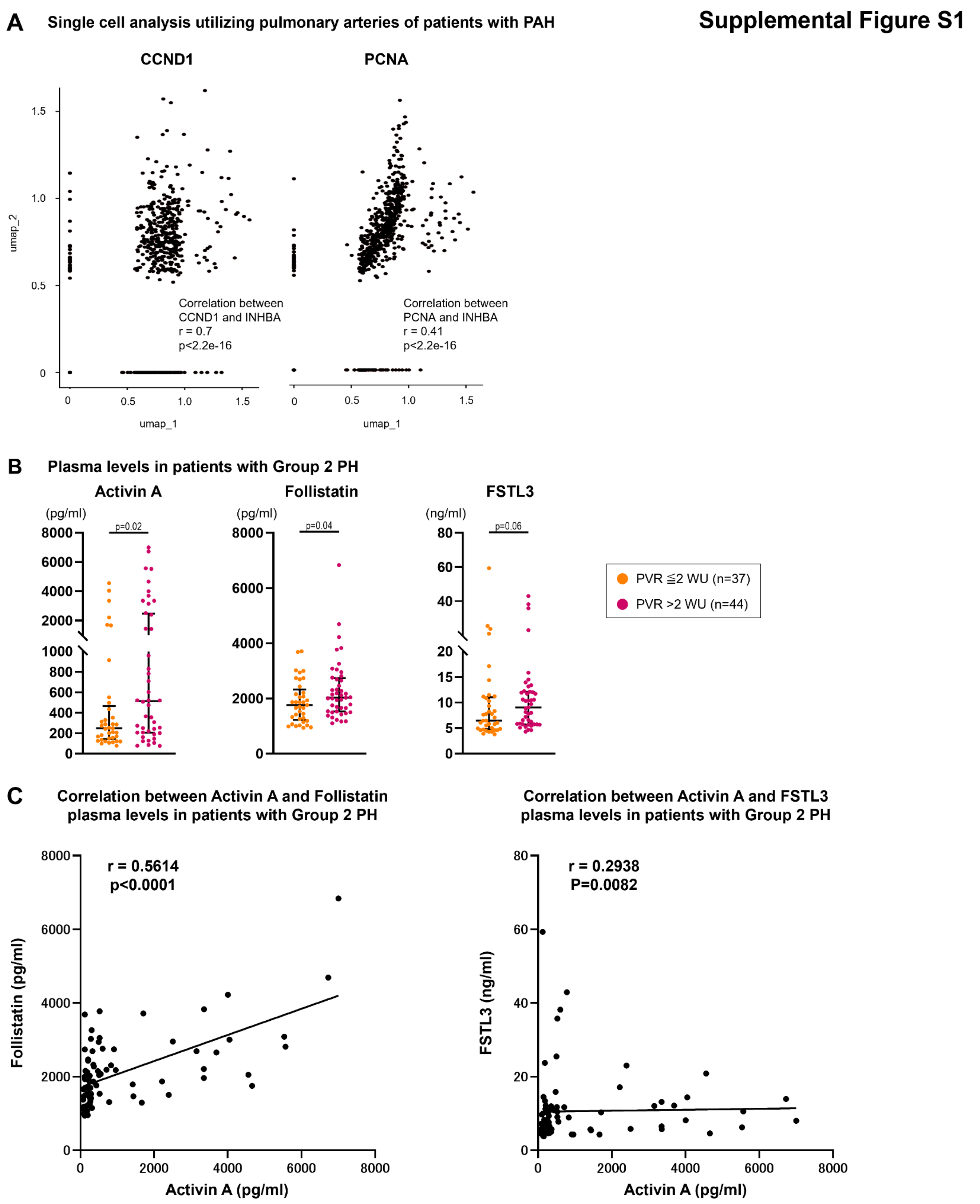

Figure S1.

(A) Feature scatter plots comparing the expression levels of INHBA, CCND1, and PCNA in the smooth muscle cluster (PAH). The plots illustrate the correlation of gene expression with statistical significance assessed using

Pearson's correlation test.
(B) Plasma levels of activin A, follistatin, and follistatin like 3 (FSTL3) in patients with Group 2 PH. Data are presented as median and IQR were analyzed using the Mann-Whitney U test.
(C) Pearson's correlation between plasma activin A and follistatin or FSTL3 levels in patients with Group 2 PH.
CCND1: cycline D1, PCNA: proliferating cell nuclear antigen.

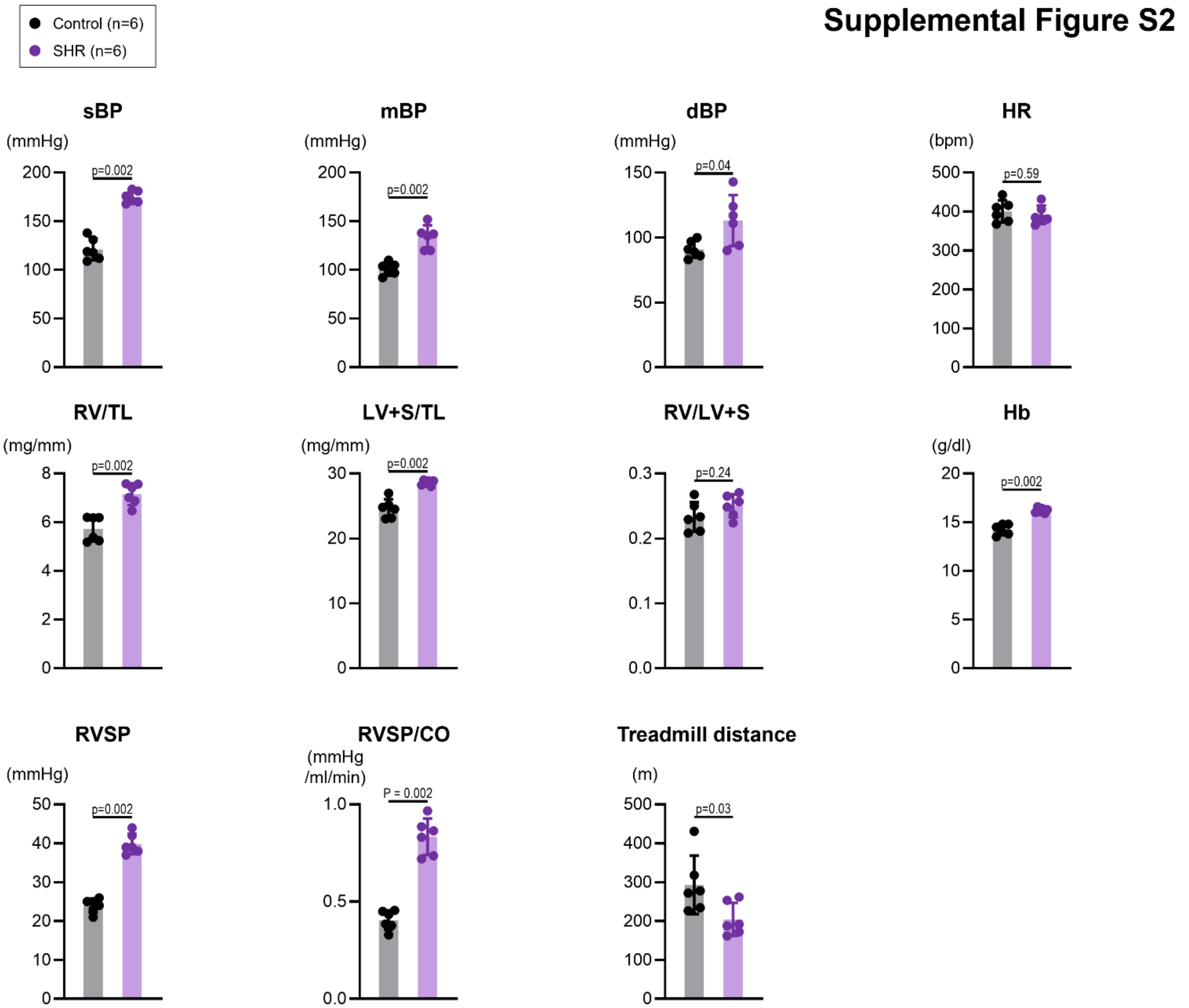

Figure S2.

Hemodynamics, ventricular hypertrophy and treadmill distance were evaluated in spontaneous hypertensive rats (SHR) and controls (n=6).

Results are expressed as mean ± SD and analyzed using the Mann-Whitney U test.

sBP, systolic blood pressure; mBP, mean blood pressure; dBP, diastolic blood pressure; HR, heart rate; RV, the weight of the right ventricle; TL, tibia length; LV+S, weight of the left ventricle plus septum; Hb, hemoglobin; RVSP, right ventricular systolic pressure.

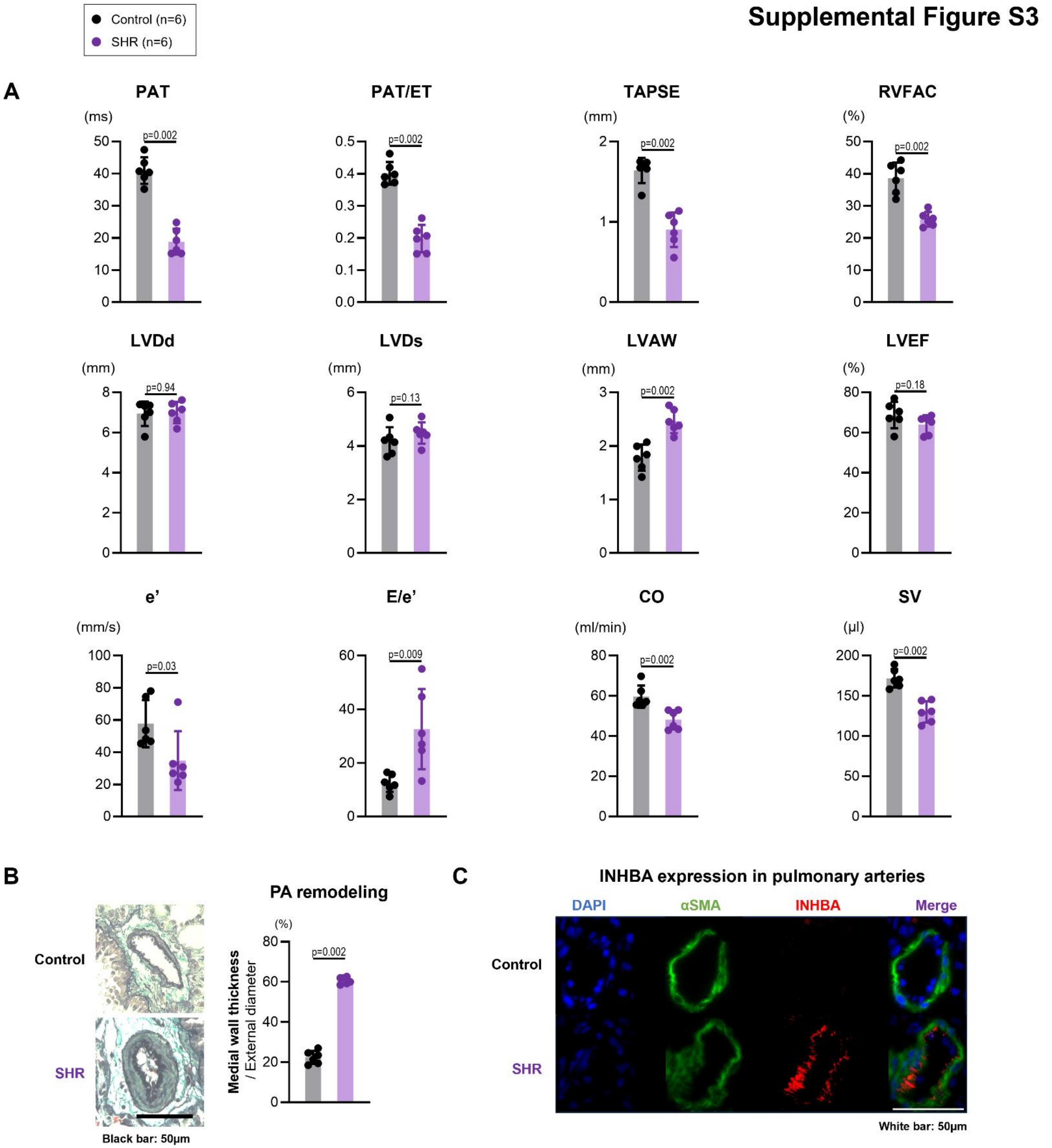

Figure S3.

(A) Echocardiography and hemodynamics were evaluated in spontaneous hypertensive rats (SHR) or control (n= 6)~~, analyzed using Mann-Whitney U test~~.

(B) Immunofluorescence images of pulmonary arteries from SHR and controls, showing αSMA (green), INHBA (red), and DAPI (blue) staining.

(C) Representative Elastica-Masson (EM) staining images and quantification of pulmonary artery remodeling in SHR and controls (n=6).

Data are presented as mean ± SD and analyzed using the Mann-Whitney U test.

PAT, pulmonary acceleration time; ET, ejection time; TAPSE, tricuspid annular plane systolic excursion; RVFAC, right ventricular fractional area change; LVDd, left ventricular diastolic diameter; LVDs, left ventricular systolic diameter; LVAW, left ventricular anterior wall thickness; LVEF, left ventricular ejection fraction; e’, peak early diastolic mitral annular velocity; E, early diastolic filling velocity; CO, cardiac output; SV, stroke volume.

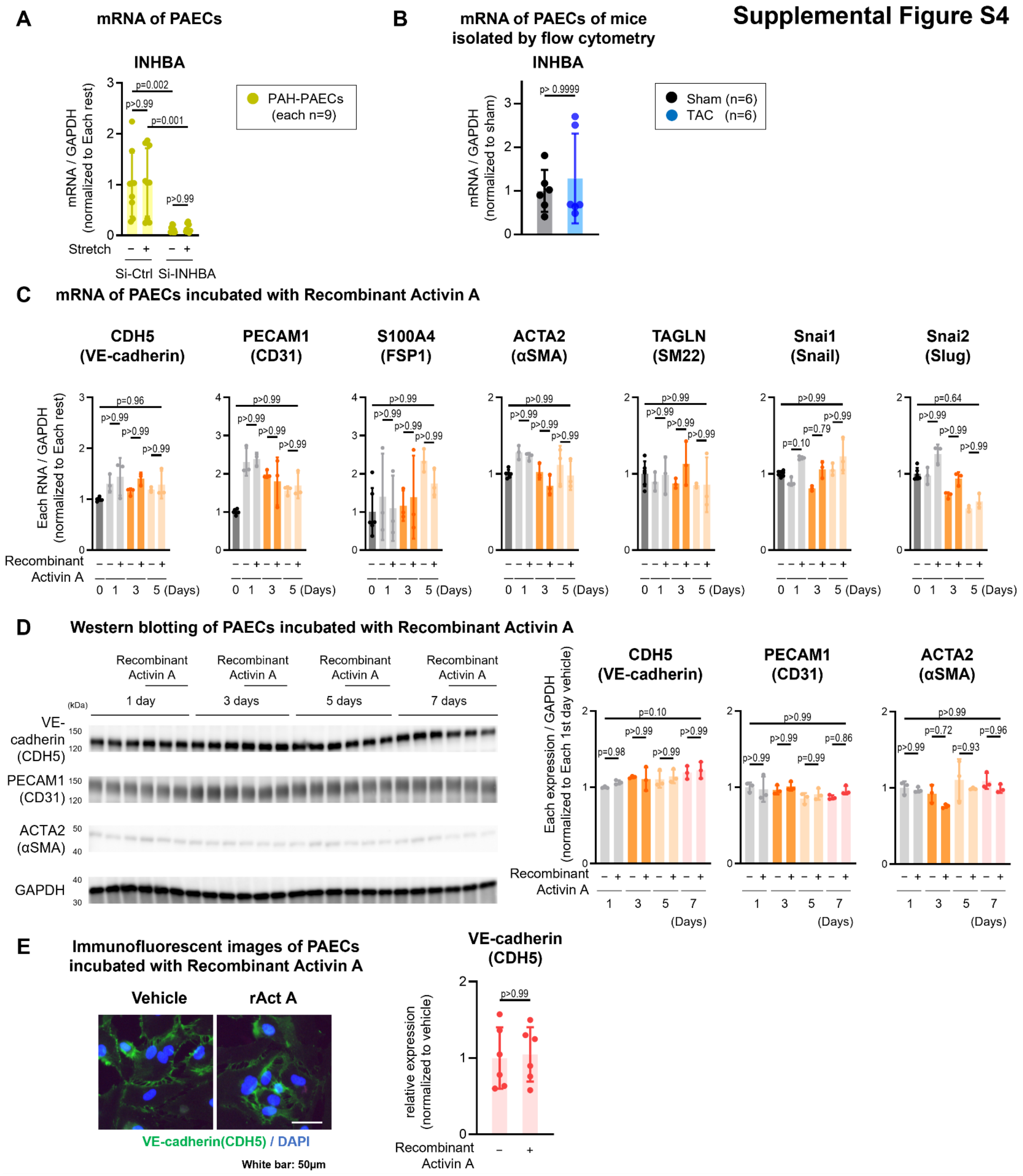

Figure S4.

(A) mRNA expression of INHBA in cultured PAECs of patients with PAH stretched for 24 h with si-INHBA or si-Ctrl (each n=9).

(**B**) INHBA expression in PAECs (CD45^{-}/CD326^{-}/CD31^{+} cells) isolated from the lungs of TAC or sham mice by flow cytometer (n=6).

(C) mRNA expression levels of CDH5, PECAM1, S100A4, ACTA2, TAGLN, Snail1, and Snail2 in PAECs

from individuals without PH treated with recombinant activin A (100 ng/ml) for 1, 3, or 5 days (n=3). **(D)** Representative western blots and quantification of VE-cadherin, PECAM1, and αSMA in PAECs from individuals without PH treated with recombinant activin A (100 ng/ml) for 1, 3, 5, or 7 days (n=3). **(E)** Representative images and quantification of VE-cadherin (green), αSMA (red), and DAPI (blue) expression in PAECs from individuals without PH treated with recombinant activin A (100 ng/ml) for 7 days (n=6). Data are presented as mean ± SD. (A and D) Comparisons between each group were analyzed using two-way ANOVA followed by Tukey's HSD test. (C) Data are analyzed using the Kruskal-Wallis test followed by Dunn's test or (B and E) using the Mann-Whitney U test. Data are presented as mean ± SD. PAECs: pulmonary artery endothelial cells.

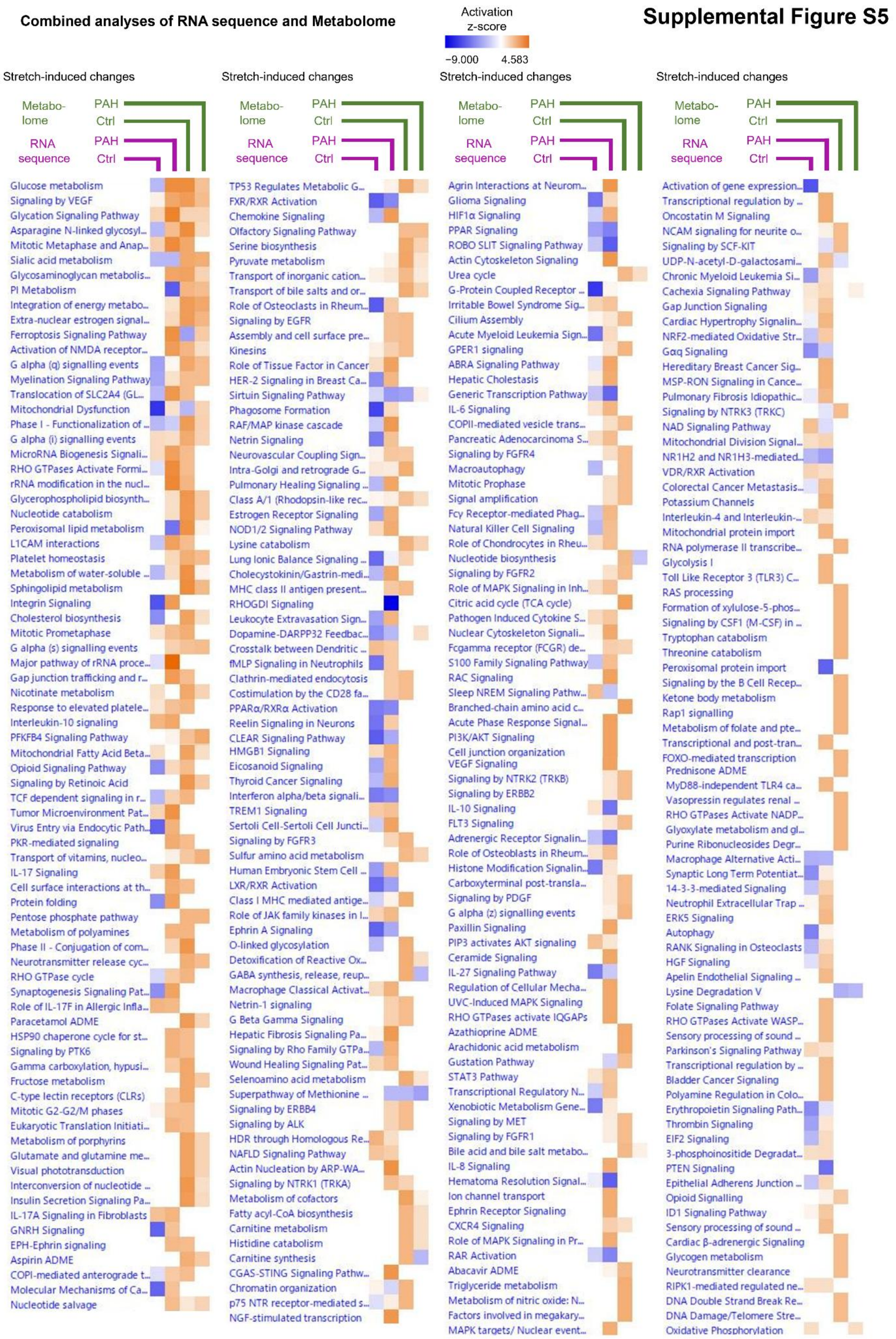

Figure S5.

Heatmap of stretch induced changes analyzed by RNA sequence and metabolome analyses with cultured PAMSCs stretched for 24 h of individuals without PH and individuals with PAH.

Supplemental Figure S6

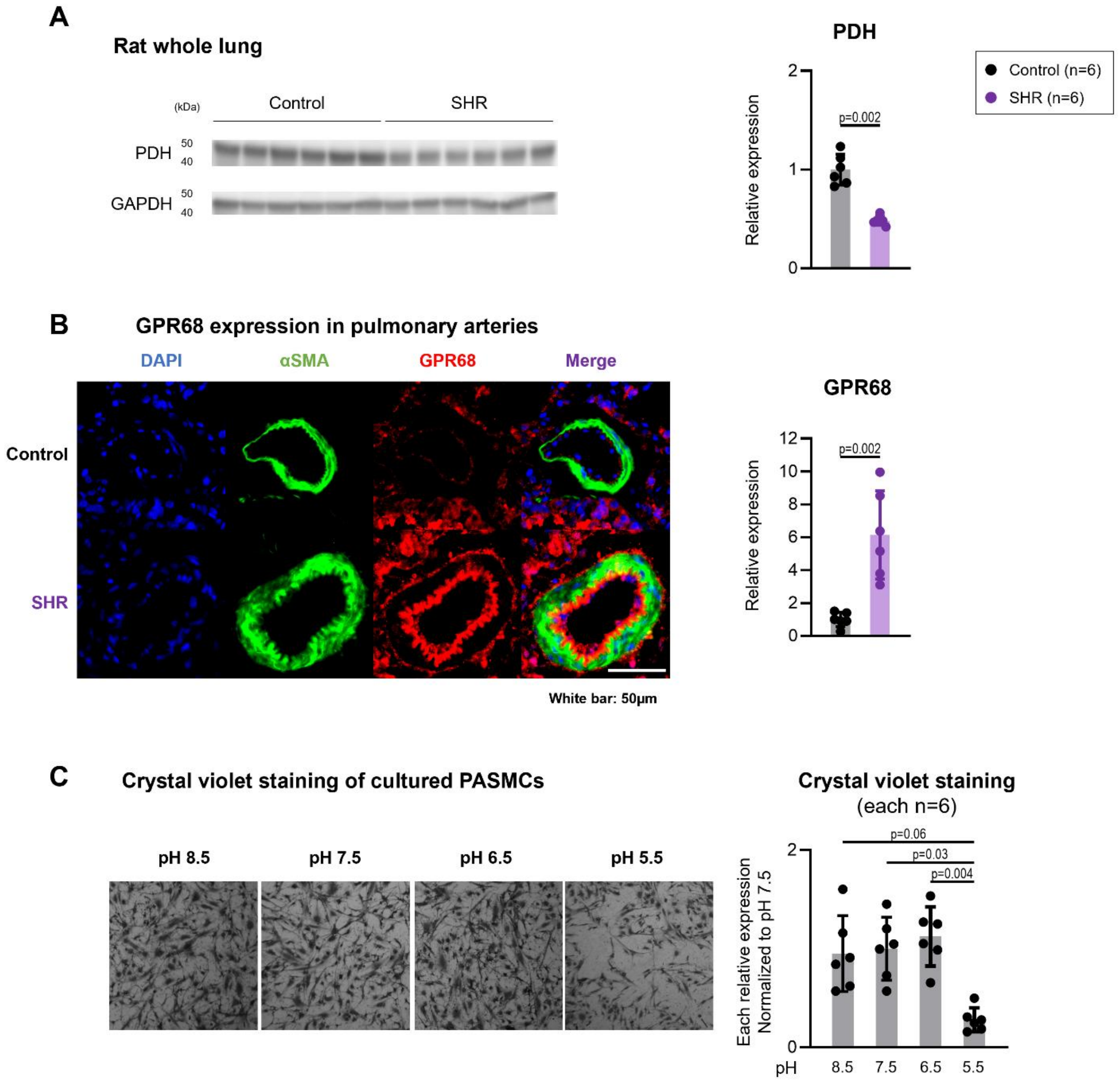

Figure S6.
(**A**) Representative western blots and quantification of PDH and GAPDH expression in the whole lungs from SHR (n=6).
(**B**) Representative images and quantification of αSMA (green), GPR68 (red), and DAPI (blue) expression in the pulmonary arteries of SHR and control, analyzed using the Mann-Whitney U test (n=6).
(C) Crystal violet staining of cultured PASMCs from non-PH individuals incubated in DMEM with adjustment of pH to 8.5, 7.5, 6.5, or 5.5 (n=6).
Results are expressed as mean ± SD and (A and B) analyzed using the Mann-Whitney U test or (C) using the Kruskal-Wallis test followed by Dunn's test

Supplemental Figure S7

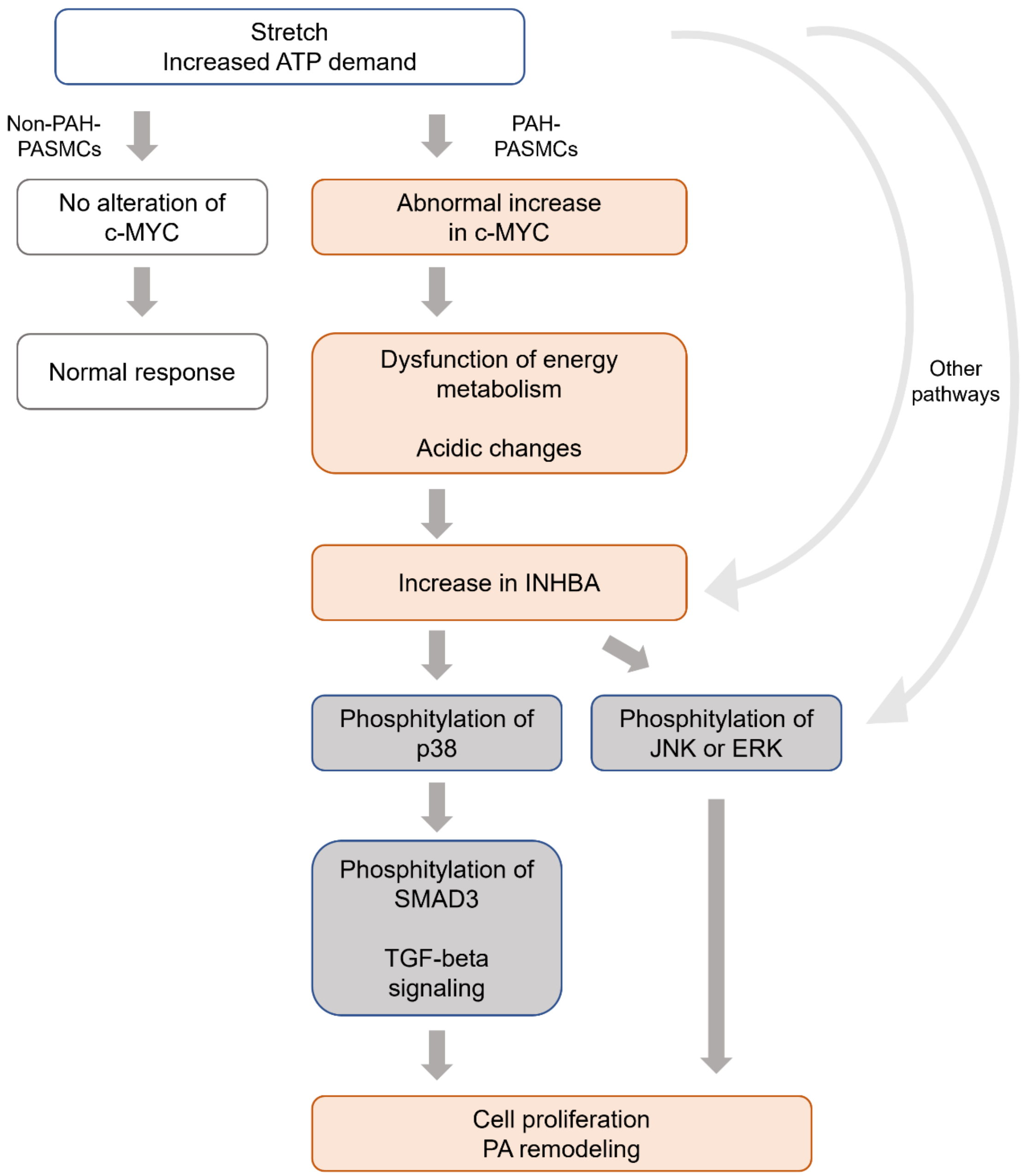

Figure S7.
Schematic diagram represents the signaling pathway we propose based on this study.

Supplemental Figure S8

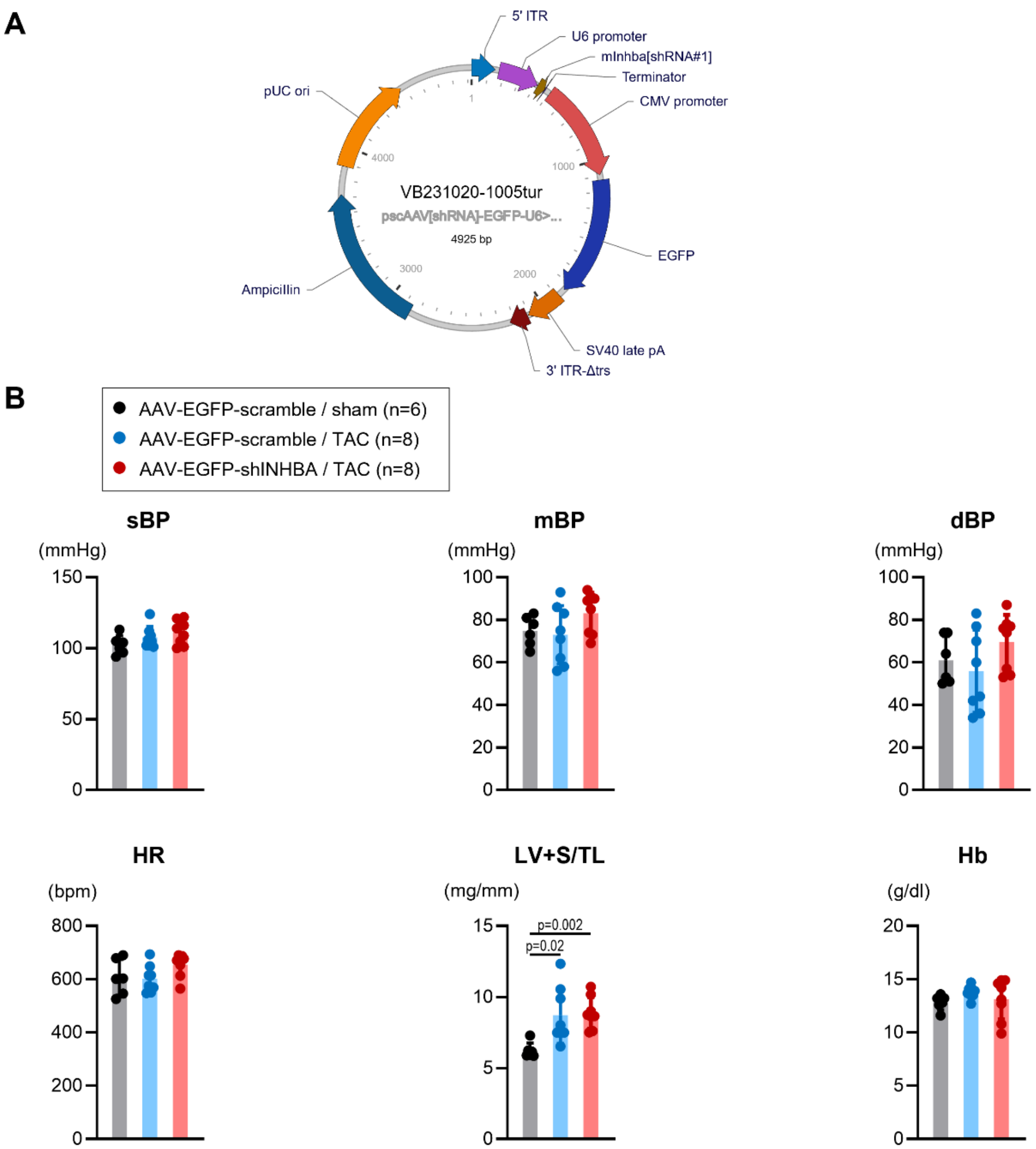

Figure S8.

(A) Schematic images of adeno-associated virus 6 vector including eGFP and cytomegalovirus promoter for mice.

(B) Hemodynamics, ventricular hypertrophy and hemogulobin were evaluated in TAC mice treated with AAV-shINHBA or AAV-scramble via intratracheal instillation (n=6–8).

Results were expressed as mean ± SD and analyzed using Kruskal-Wallis test followed by Dunn's test.

sBP, systolic blood pressure: mBP, mean blood pressure: dBP, diastolic blood pressure: HR, heart rate: LV+S, the weight of the left ventricle plus septum: TL, tibial length: Hb, hemoglobin.

Supplemental Figure S9

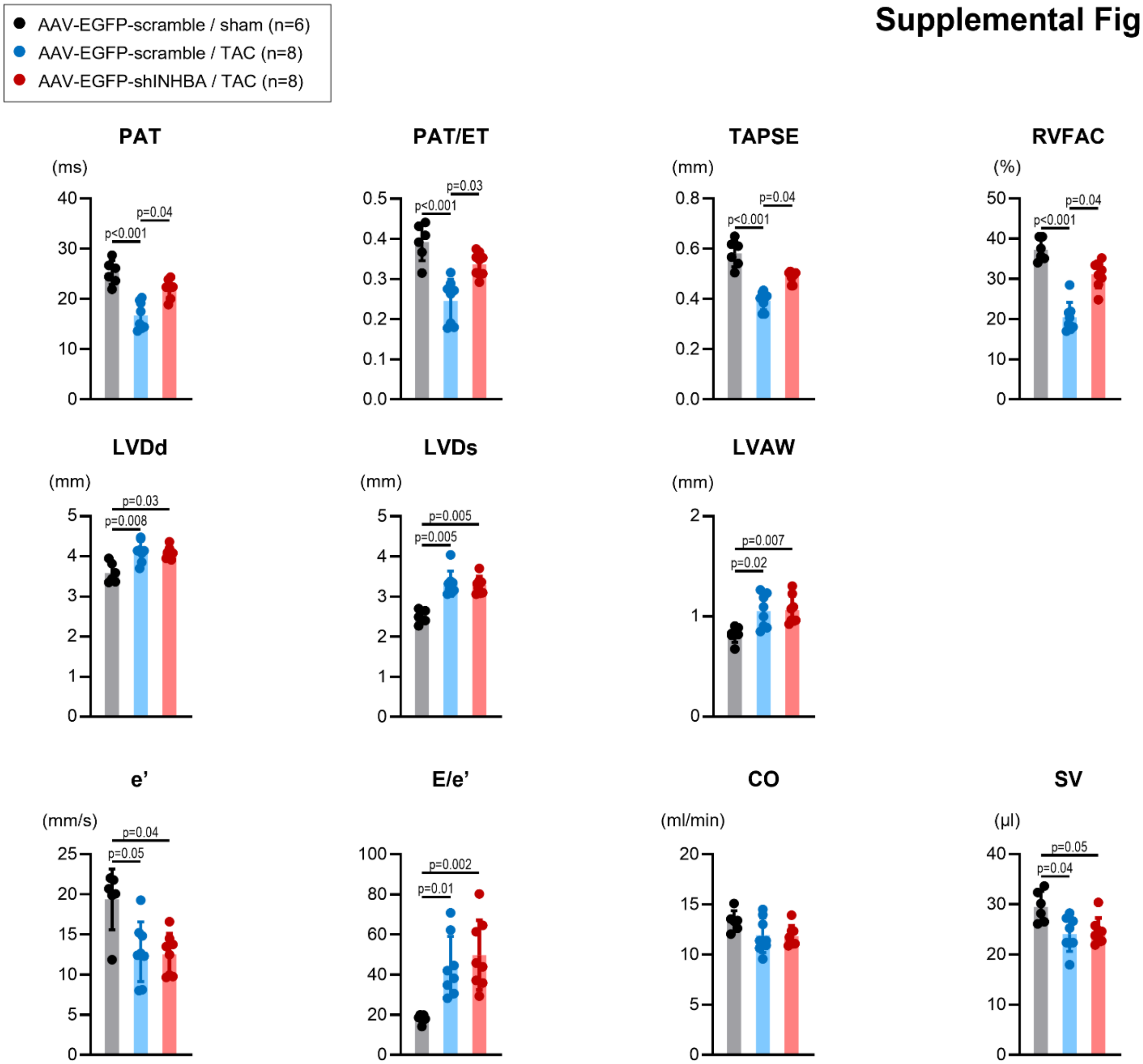

Figure S9.

Echocardiography and hemodynamics were evaluated in TAC mice treated with AAV-shINHBA or AAV-scramble via intratracheal instillation (n=6–8).

Results were expressed as mean ± SD and analyzed using the Kruskal-Wallis test followed by Dunn’s test.

PAT, pulmonary acceleration time; ET, ejection time; TAPSE, tricuspid annular plane systolic excursion; RVFAC, right ventricular fractional area change; LVDd, left ventricular diastolic diameter; LVDs, left ventricular systolic diameter; LVAW, left ventricular anterior wall thickness; e’, e’, peak early diastolic mitral annular velocity; E, early diastolic filling velocity; CO, cardiac output; SV, stroke volume.

Supplemental Figure S10

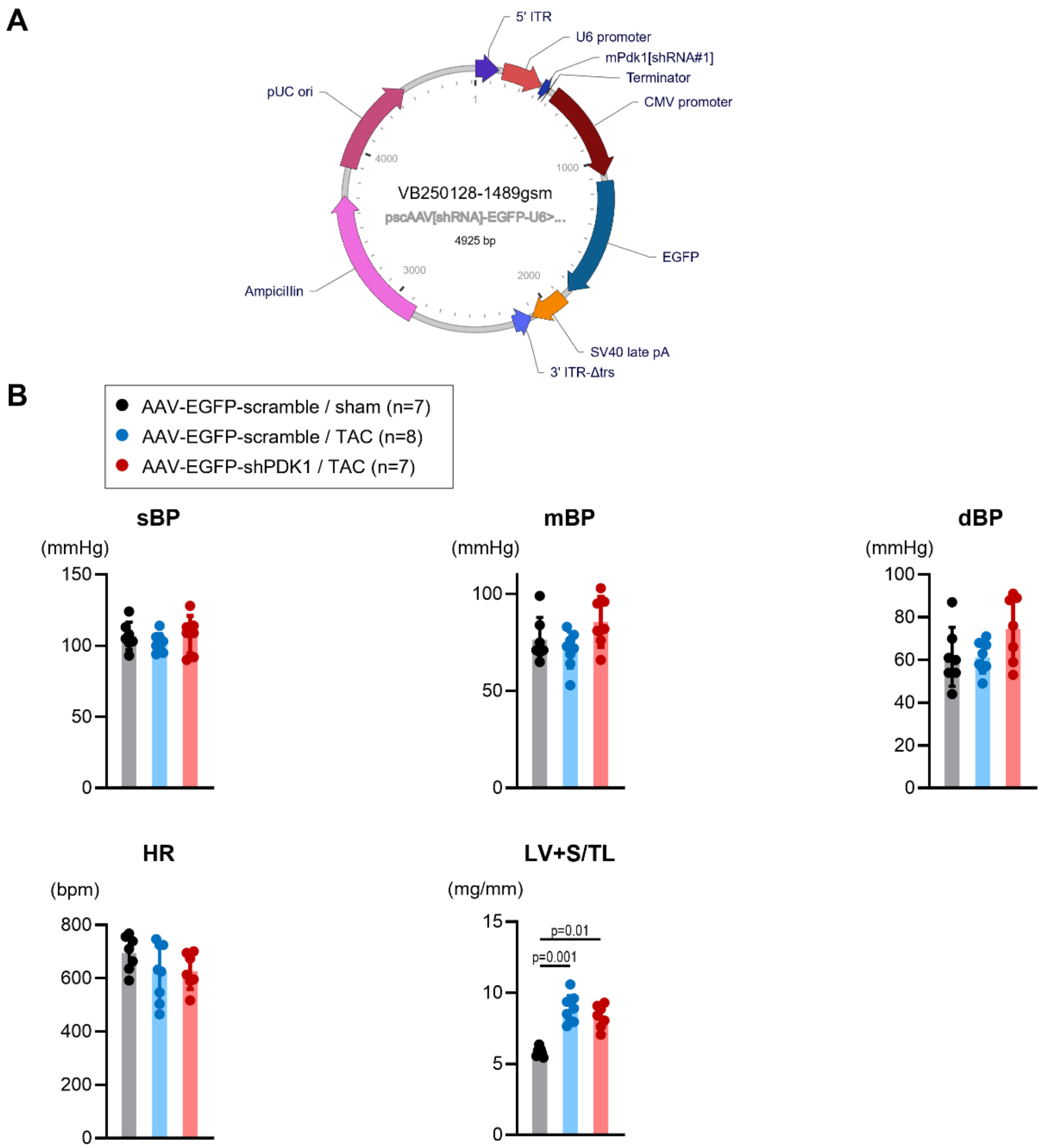

Figure S10.
(**A**) Schematic images of adeno-associated virus 6 vector including eGFP and cytomegalovirus promoter for mice.
(**B**) Hemodynamics and ventricular hypertrophy were evaluated in TAC mice treated with AAV-shPDK1 or AAV-scramble via intratracheal instillation (n=7–8).
Results were expressed as mean ± SD and analyzed using the Kruskal-Wallis test followed by Dunn's test.
sBP, systolic blood pressure; mBP, mean blood pressure; dBP, diastolic blood pressure; HR, heart rate; LV+S, the weight of the left ventricle plus septum; TL, tibial length.

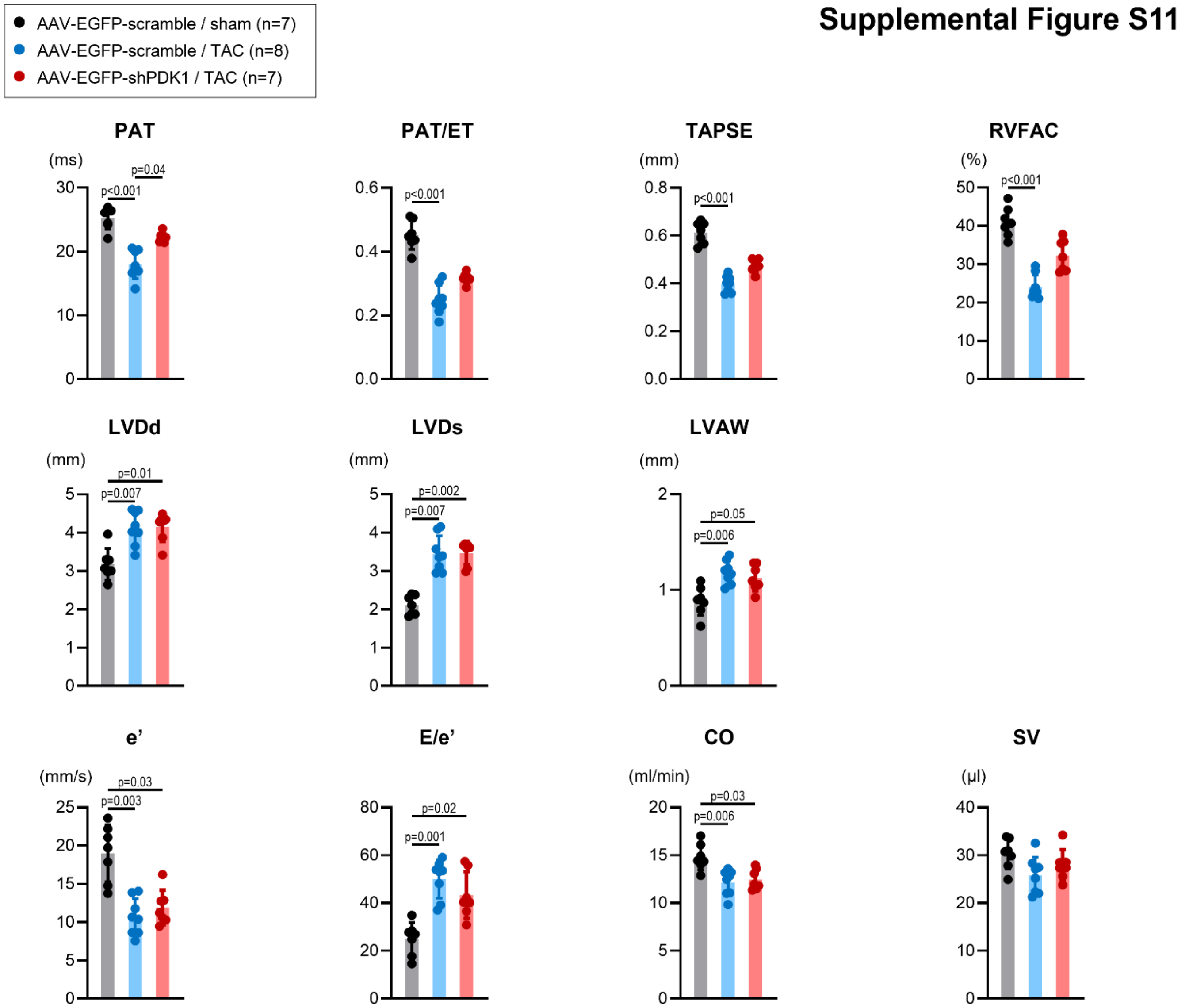

Figure S11.

Echocardiography and hemodynamics were evaluated in TAC mice treated with AAV-shPDK1 or AAV-scramble via intratracheal instillation (n=7–8).

Results are expressed as mean ± SD and analyzed using the Kruskal-Wallis test followed by Dunn's test.

PAT, pulmonary acceleration time; ET, ejection time; TAPSE, tricuspid annular plane systolic excursion; RVFAC, right ventricular fractional area change; LVDd, left ventricular diastolic diameter; LVDs, left ventricular systolic diameter; LVAW, left ventricular anterior wall thickness; e', mitral annular tissue Doppler; E, mitral inflow; CO, cardiac output; SV, stroke volume.

Major Resources Table

In order to allow validation and replication of experiments, all essential research materials listed in the Methods should be included in the Major Resources Table below. Authors are encouraged to use public repositories for protocols, data, code, and other materials and provide persistent identifiers and/or links to repositories when available. Authors may add or delete rows as needed.

Animals (in vivo studies)

Species	Vendor or Source	Background Strain	Sex	Persistent ID / URL
Wild-type C57BL/6	Japan SLC Inc.	C57BL/6J	Male	http://www.jslc.co.jp/english/animals/mouse.php
spontaneously hypertensive rat	Japan SLC Inc.	SHR/Izm	Male	http://www.jslc.co.jp/animals/rat.php#rat-cat-03
Wistar Kyoto rat	Japan SLC Inc.	WKY/Izm	Male	http://www.jslc.co.jp/animals/rat.php#rat-cat-03

Genetically Modified Animals

	Species	Vendor or Source	Background Strain	Other Information	Persistent ID / URL
**Parent - Male**					
**Parent - Female**					

Antibodies

Target antigen	Vendor or Source	Catalog #	Working concentration	Lot # (preferred but not required)	Persistent ID / URL
**Western blot**					
INHBA	Abcam	ab128958	1:1000 (0.1 µg/ml)		https://www.abcam.co.jp/products/primary-antibodies/inhibin-beta-a-antibody-epr27852-ab128958.html
pSMAD3	Abcam	ab52903	1:2000 (0.25 µg/ml)		https://www.abcam.co.jp/products/primary-antibodies/smad3-ps423425--smad5-ps463465---smad1-ps463465--smad2-ps465467-antibody-ep823y-ab52903.html
SMAD2/3	Cell Signaling Technology	8685	1:1000		https://www.cellsignal.jp/products/primary-antibodies/smad2-3-d7g7-xp-rabbit-mab/8685
pJNK	Cell Signaling Technology	4668	1:1000		https://www.cellsignal.jp/products/primary-antibodies/phospho-sapk-jnk-thr183-tyr185-81e11-rabbit-mab/4668
tJNK	Cell Signaling Technology	9252	1:1000		https://www.cellsignal.jp/products/primary-antibodies/sapk-jnk-antibody/9252
pERK1/2	Cell Signaling Technology	9101	1:1000		https://www.cellsignal.jp/products/primary-antibodies/phospho-p44-42-mapk-erk1-2-thr202-tyr204-antibody/9101

tERK1/2	Cell Signaling Technology	9102	1:1000		https://www.cellsignal.jp/products/primary-antibodies/p44-42-mapk-erk1-2-antibody/9102
p-p38	Cell Signaling Technology	9211	1:1000		https://www.cellsignal.jp/products/primary-antibodies/phospho-p38-mapk-thr180-tyr182-antibody/9211
p38	Cell Signaling Technology	9212	1:1000		https://www.cellsignal.jp/products/primary-antibodies/p38-mapk-antibody/9212
c-MYC	Cell Signaling Technology	9402	1:1000		https://www.cellsignal.jp/products/primary-antibodies/c-myc-antibody/9402
PDK1	Cell Signaling Technology	3062	1:1000		https://www.cellsignal.jp/products/primary-antibodies/pdk1-antibody/3062
PDH	Cell Signaling Technology	2784	1:1000		https://www.cellsignal.jp/products/primary-antibodies/pyruvate-dehydrogenase-antibody/2784
GAPDH	Cell Signaling Technology	2118	1:1000		https://www.cellsignal.jp/products/primary-antibodies/gapdh-14c10-rabbit-mab/2118
VE-cadherin	Abcam	ab33168	1:1000 (0.7 µg/ml)		https://www.abcam.co.jp/products/primary-antibodies/ve-cadherin-antibody-intercellular-junction-marker-ab33168.html
ACTA2	Abcam	ab5694	1:1000 (0.2 µg/ml)		https://www.abcam.co.jp/products/primary-antibodies/alpha-smooth-muscle-actin-antibody-ab5694.html
PECAM1	Proteintech	11265-1-AP	1:1000 (0.013 µg/ml)		https://www.ptglab.co.jp/products/PECAM1-Antibody-11265-1-AP.htm
Anti-rabbit IgG HRP-linked Antibody	Cell Signaling Technology	7074	1:5000		https://www.cellsignal.com/products/secondary-antibodies/anti-rabbit-igg-hrp-linked-antibody/7074?srsltid=AfmBOopsrhGg-MWTab6hbrivxo3f9Ra0LNqQqMnkA6QGVUh-3uJbZkuS
**IF**					
Activin A	R&D Systems	AF338	1:40 (5 µg/ml)		https://www.rndsystems.com/products/human-mouse-rat-activin-a-betaa-subunit-antibody_af338
αSMA-cy3	Sigma-Aldrich	C6198	1:100 (10-15 µg/ml)		https://www.sigmaaldrich.com/JP/ja/product/sigma/c6198
αSMA-488	Cell Signaling Technology	46469	1:100		https://www.cellsignal.jp/products/antibody-conjugates/a-smooth-muscle-actin-1a4-mouse-mab-alexa-fluor-488-conjugate/46469

pSMAD3	Abcam	ab52903	1:250<br>(2 µg/ml)		https://www.abcam.co.jp/products/primary-antibodies/smad3-ps423425--smad5-ps463465---smad1-ps463465--smad2-ps465467-antibody-ep823y-ab52903.htmlsmad1-ps463465--smad2-ps465467-antibody-ep823y-ab52903.html
GPR68	Thermo Fisher	720277	1:250<br>(2 µg/ml)		https://www.thermofisher.com/antibody/product/GPR68-Antibody-Polyclonal/720277
CD31	Abcam	ab28364	1:100<br>(0.13 µg/ml)		https://www.abcam.co.jp/products/primary-antibodies/cd31-antibody-ab28364.html
VE-cadherin	Abcam	ab33168	1:400<br>(1.75 µg/ml)		https://www.abcam.co.jp/products/primary-antibodies/ve-cadherin-antibody-intercellular-junction-marker-ab33168.html
αSMA	Sigma-Aldrich	a5228	1:200<br>(~10 µg/ml)		https://www.sigmaaldrich.com/JP/ja/product/sigma/a5228?srsltid=AfmBOoqDPPnQ26ObvN6eCgCFWKOAVxfCGf89p63ZwK7Ft7KBnD1-0Uf_
DAPI	Vector Laboratories	H-1500-10	50µl/slide		https://vectorlabs.com/products/vectashield-hardset-with-dapi/?srsltid=AfmBOoqB7I6Kqc-PdBsLvr73-obZAxTzos3fn4RLdIhftWZDWvGHj_qw
**Flow cytometry**					
PE-conjugated Rat anti-Mouse CD31	BD	561073	1:40<br>(5 µg/ml)		https://www.bdbiosciences.com/en-ie/products/reagents/flow-cytometry-reagents/research-reagents/single-color-antibodies-ruo/pe-rat-anti-mouse-cd31.561073?tab=product_details
APC-conjugated Rat anti-Mouse CD45	BD	559864	1:40<br>(5 µg/ml)		https://www.bdbiosciences.com/en-ie/products/reagents/flow-cytometry-reagents/research-reagents/single-color-antibodies-ruo/apc-rat-anti-mouse-cd45.559864?tab=product_details
APC-conjugated anti-Mouse CD326	BD	563478	1:40<br>(5 µg/ml)		https://www.bdbiosciences.com/en-dk/products/reagents/flow-cytometry-reagents/research-reagents/single-color-antibodies-ruo/apc-rat-anti-mouse-cd326.563478?tab=product_details
SYTOX™ Blue Dead Cell Stain, for flow cytometry	Thermo Fisher	S34857	1 µM		https://www.thermofisher.com/order/catalog/product/S34857

DNA/cDNA Clones

Clone Name	Sequence	Source / Repository	Persistent ID / URL
pscAAV[shRNA]-EGFP-U6>mInhba[shRNA#1]	TCTGGCTATCACGCCAATTAT	VectorBuilder	https://www.vectorbuilder.jp/vector/VB231020-1005tur.html
pscAAV[shRNA]-EGFP-U6>mPdk1[shRNA#1]	CGGCTTTGTGATTTGTATTAT	VectorBuilder	https://www.vectorbuilder.jp/vector/VB250128-1489gsm.html
pAAV[shRNA]-EGFP-U6>Scramble_shRNA	CCTAAGGTTAAGTCGCCCTCG	VectorBuilder	https://www.vectorbuilder.jp/vector/VB010000-0023jze.html

Cultured Cells

Name	Vendor or Source	Sex (F, M, or unknown)	Persistent ID / URL
Pulmonary artery smooth muscle cells	Cultured from patients with PAH during lung transplantation and from non-PH patients undergoing thoracic surgery for lung cancer.	M, F	N/A
Pulmonary artery endothelial cells	Cultured from patients with PAH during lung transplantation and from non-PH patients undergoing thoracic surgery for lung cancer.	M, F	N/A

Data & Code Availability

Description	Source / Repository	Persistent ID / URL
Single cell RNA-sequencing data	NCBI BioProject/SRA	https://www.ncbi.nlm.nih.gov/bioproject/PRJNA989574
RNA-sequencing data	NCBI BioProject/SRA	https://www.ncbi.nlm.nih.gov/bioproject/PRJNA864822

Other

Description	Source / Repository	Catalog #	Persistent ID / URL
Lactate Assay Kit	Sigma-Aldrich	MAK064, MK570	https://www.sigmaaldrich.com/JP/ja/product/sigma/mak570?srsltid=AfmBOorVWqxSKclE3x3zq5IYQ2SK3zJo8W2a67M6UqCBxGyfmpo0aVdp
Pyruvate Assay Kit	Sigma-Aldrich	MAK071	https://www.sigmaaldrich.com/JP/ja/product/sigma/mak071?srsltid=AfmBOorsx4UlfwexSHfLLV6cLufsyGdZpw4loHFHcRD_gr5fvVLMxfeF
Acetyl-Coenzyme A Kit	Sigma-Aldrich	MAK039	https://www.sigmaaldrich.com/JP/ja/product/sigma/mak039?srsltid=AfmBOopabPE3JWo_A1H4rHM4-yL5eVxKQbuLL0a74RX0HkOaEVPpAMq4

Cell Proliferation ELISA, BrdU	Roche	11647229001	https://www.sigmaaldrich.com/JP/ja/product/roche/11647229001
Recombinant Activin A	R&D Systems	11348-AC	https://www.rndsystems.com/products/recombinant-human-activin-a-cho-derived-protein-cf_11348-ac
PD98059	Cell Signaling Technology	9900	https://www.cellsignal.jp/products/activators-inhibitors/pd98059/9900
SP600125	Tokyo Chemical Industry	P3160	https://www.tcichemicals.com/JP/ja/p/P3160
SB203580	Tokyo Chemical Industry	F0864	https://www.tcichemicals.com/JP/ja/p/F0864
Crystal violet solution	Sigma-Aldrich	V5265	https://www.sigmaaldrich.com/JP/ja/product/sigma/v5265?srsltid=AfmBOoo00nSV1tswVKxMupK_nhUtDd6zqoWA4QGSUrbyNO0i6MgDrKVg
Human/Mouse/Rat Activin A ELISA kit	R&D Systems	DAC00B	https://www.rndsystems.com/products/human-mouse-rat-activin-a-quantikine-elisa-kit_dac00b
Human Follistatin ELISA kit	R&D Systems	DFN00	https://www.rndsystems.com/products/human-follistatin-quantikine-elisa-kit_dfn00
Human FLRG ELISA kit	R&D Systems	DFLRG0	https://www.rndsystems.com/products/human-flrg-quantikine-elisa-kit_dflrg0

ARRIVE GUIDELINES

The ARRIVE guidelines (https://arriveguidelines.org/) are a checklist of recommendations to improve the reporting of research involving animals. Key elements of the study design should be included below to better enable readers to scrutinize the research adequately, evaluate its methodological rigor, and reproduce the methods or findings.

Study Design

TAC

Groups	Sex	Age	Number (prior to experiment)	Number (after termination)	Littermates (Yes/No)	Other description
Sham	M	10 weeks	6	6	NO	Control group
TAC	M	10 weeks	8	6	NO	2:died due to TAC operation

SHR

Groups	Sex	Age	Number (prior to experiment)	Number (after termination)	Littermates (Yes/No)	Other description
WKY	M	18 weeks	6	6	NO	Control group
SHR	M	18 weeks	6	6	NO	

TAC with AAV-shINHBA

Groups	Sex	Age	Number (prior to experiment)	Number (after termination)	Littermates (Yes/No)	Other description
Group 1 (Sham operation with AAV-scramble)	M	10 weeks	8	6	NO	Control group 2:died due to administration of AAV.
Group 2 (TAC operation with AAV-scramble)	M	10 weeks	12	8	NO	3:died due to TAC operation. 1:died due to administration of AAV.
Group 3 (TAC operation with AAV-shINHBA)	M	10 weeks	12	8	NO	2:died due to TAC operation. 1:died due to administration of AAV. 1:died due to anesthesia during echocardiographic examination

TAC with AAV-shPDK1

Groups	Sex	Age	Number (prior to experiment)	Number (after termination)	Littermates (Yes/No)	Other description
Group 1 (Sham operation with AAV-scramble)	M	10 weeks	8	7	NO	Control group 1:died due to administration of AAV.
Group 2 (TAC operation with AAV-scramble)	M	10 weeks	12	8	NO	2:died due to TAC operation. 2:died due to administration of AAV.
Group 3 (TAC operation with AAV-shPDK1)	M	10 weeks	12	7	NO	3:died due to TAC operation. 2:died due to administration of AAV.

Sample Size:

Statistical power of all comparisons with significant difference was calculated based on sample size, minimum effect of interest, variability (SD/means difference), and significant levels (α=0.05) using G*Power 3.1.9.7 as previously described.[2] The appropriate sample size (ie, n=6-8) was determined to achieve statistical power of >80% for all comparisons. The minimum statistical power was 82% for comparing RVSP, which was most important parameter to evaluate pulmonary hypertension in this animal experiments, of tree groups of AAV treated experiments of TAC mice, with n=6.

Inclusion Criteria

We used healthy C57BL/6 male mice at 10 weeks of age. We included mice with a transverse aortic velocity exceeding 4 m/s, as assessed by echocardiography one week after TAC operation.

Exclusion Criteria

Due to technical issues, mice that died during anesthesia or immediately after surgery were excluded from the study. Additionally, mice that exhibited severe debilitation and were unable to consume food were euthanized.

Randomization

Mice were randomly assigned to different experimental groups by alternately allocating them to each group.

Blinding

All experiments were conducted by different researchers in a blinded manner.
