# Supplementary material for: Pathogenesis of Pulmonary Artery Remodeling: TGF-Beta Signaling and Inhibin Subunit Beta A in Group 1 and 2 Pulmonary Hypertension
Source: Arterioscler Thromb Vasc Biol. 2026 Jan 22;46(3):e322506. doi: 10.1161/ATVBAHA.125.322506 (PMC12931867; doi:10.1161/ATVBAHA.125.322506)
Supplement: Supplementary file 2 [file atv-46-e322506-s002.pdf]

Figure 2

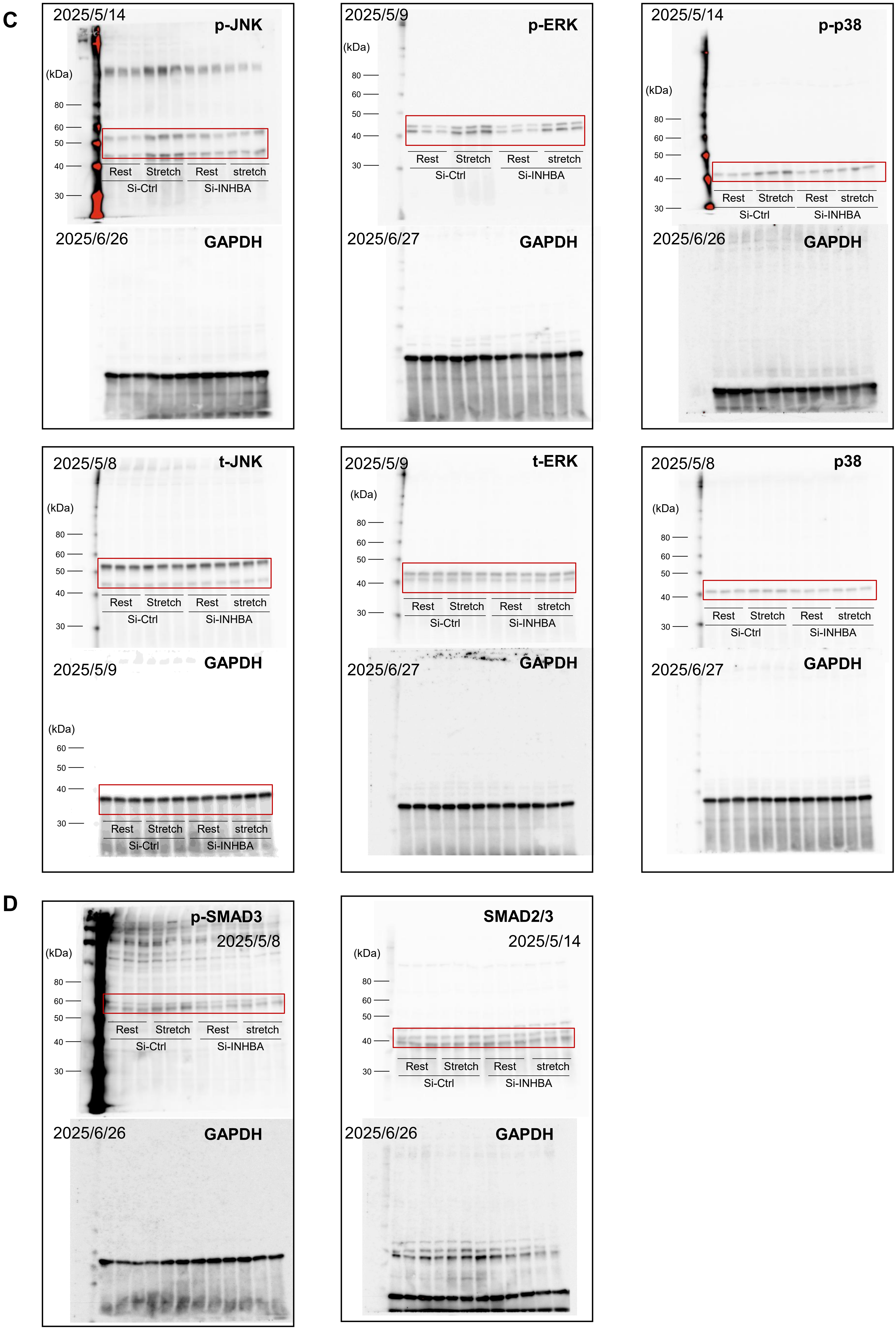
C
2025/5/14
p-JNK
(kDa)
80
60
50
40
30
Rest
Stretch
Rest
stretch
Si-Ctrl
Si-INHBA
2025/6/26
GAPDH
2025/5/9
p-ERK
2025/6/27
GAPDH
2025/5/14
p-p38
2025/6/26
GAPDH
2025/5/8
t-JNK
2025/5/9
GAPDH
2025/5/9
t-ERK
2025/6/27
GAPDH
2025/5/8
p38
2025/6/27
GAPDH
D
p-SMAD3
2025/5/8
2025/6/26
GAPDH
SMAD2/3
2025/5/14
2025/6/26
GAPDH

Figure 2

H

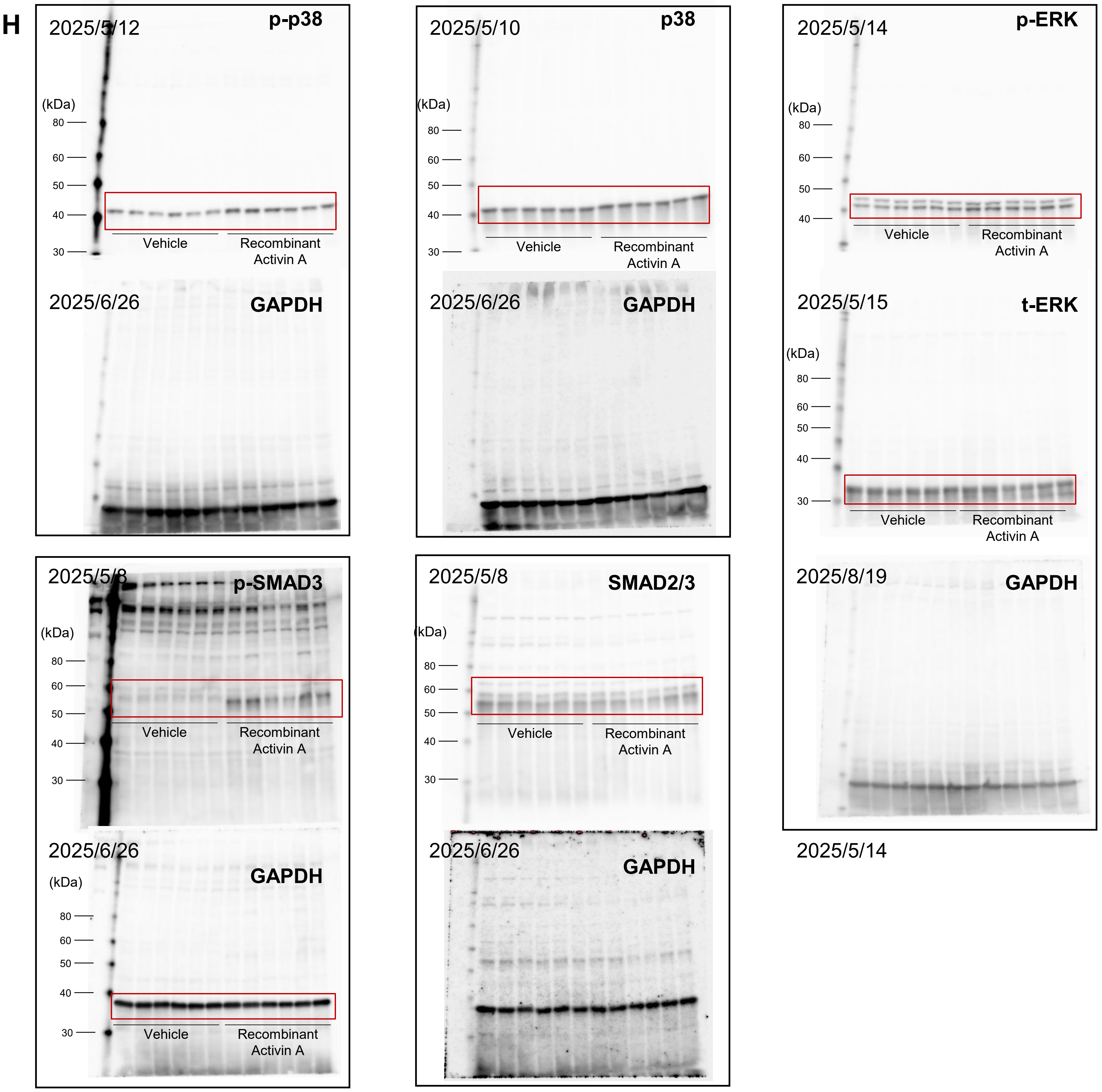

J

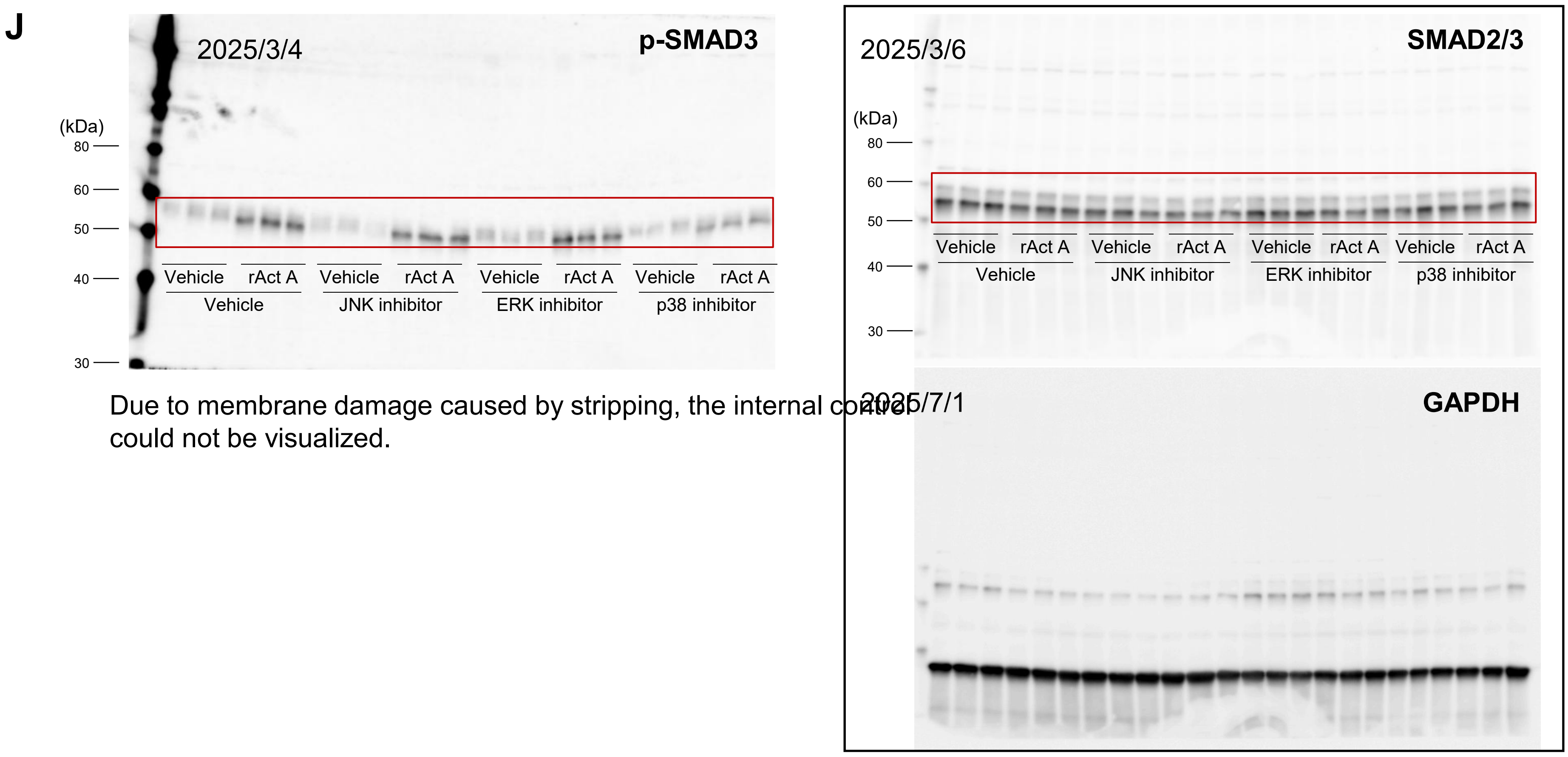

Due to membrane damage caused by stripping, the internal control could not be visualized.

B **Figure 3**

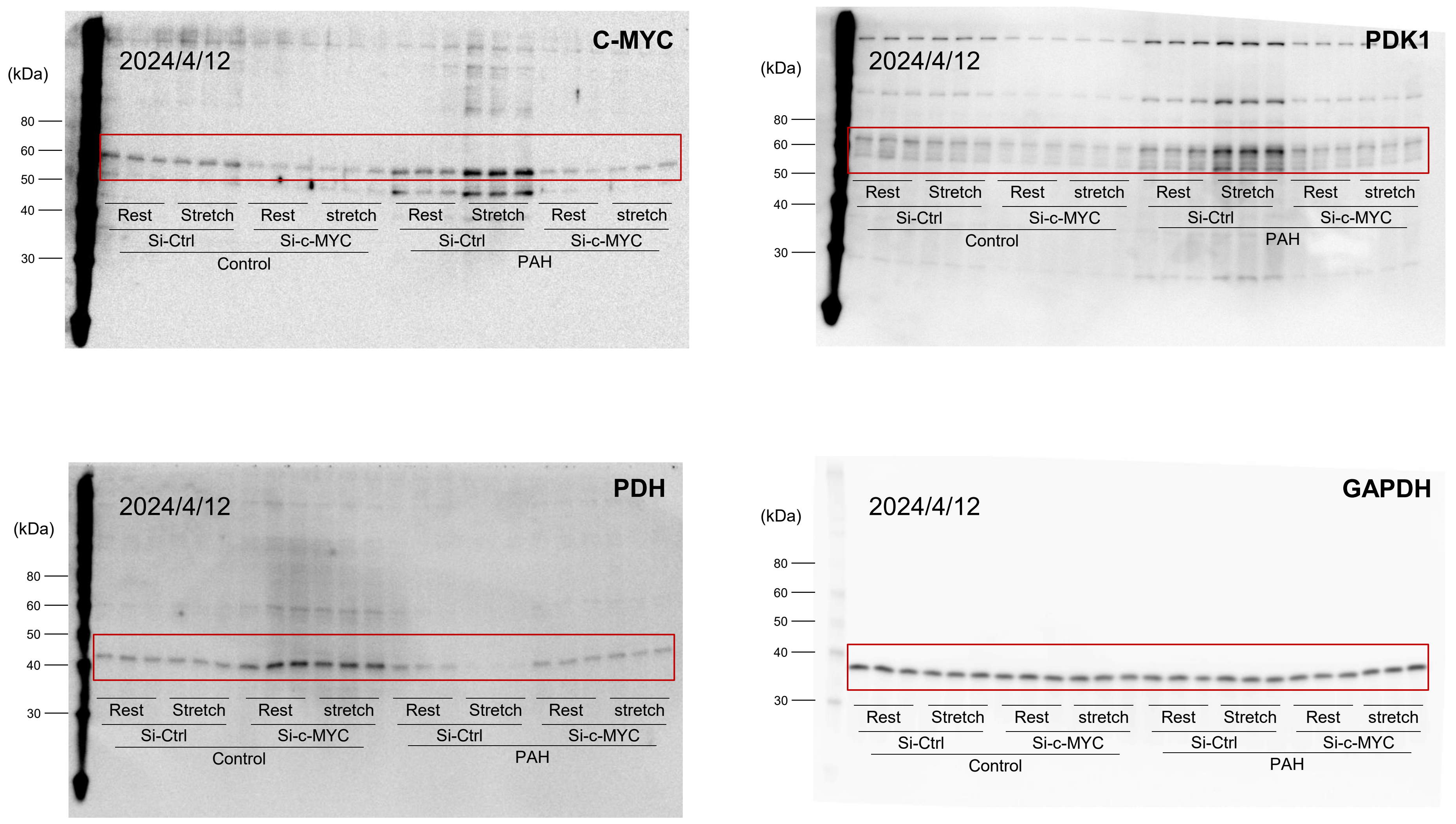

Due to membrane damage caused by stripping, the internal controls could not be visualized.

Figure 4

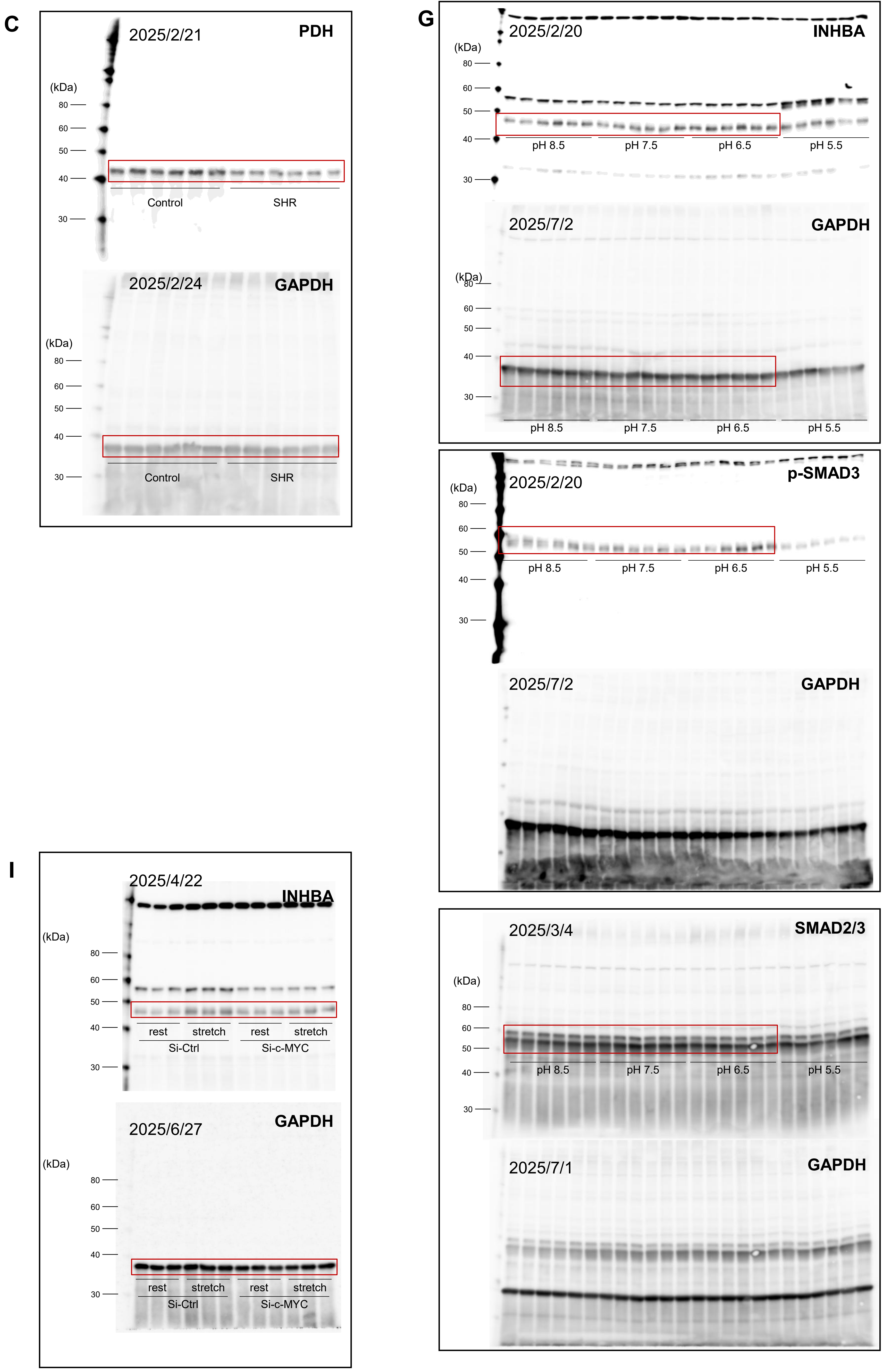

C
2025/2/21
PDH
(kDa)
80
60
50
40
30
Control
SHR
2025/2/24
GAPDH
(kDa)
80
60
50
40
30
Control
SHR
G
2025/2/20
INHBA
(kDa)
80
60
50
40
30
pH 8.5
pH 7.5
pH 6.5
pH 5.5
2025/7/2
GAPDH
(kDa)
80
60
50
40
30
pH 8.5
pH 7.5
pH 6.5
pH 5.5
2025/2/20
p-SMAD3
(kDa)
80
60
50
40
30
pH 8.5
pH 7.5
pH 6.5
pH 5.5
2025/7/2
GAPDH
2025/3/4
SMAD2/3
(kDa)
80
60
50
40
30
pH 8.5
pH 7.5
pH 6.5
pH 5.5
2025/7/1
GAPDH
I
2025/4/22
INHBA
(kDa)
80
60
50
40
30
rest
stretch
rest
stretch
Si-Ctrl
Si-c-MYC
2025/6/27
GAPDH
(kDa)
80
60
50
40
30
rest
stretch
rest
stretch
Si-Ctrl
Si-c-MYC

Figure 4

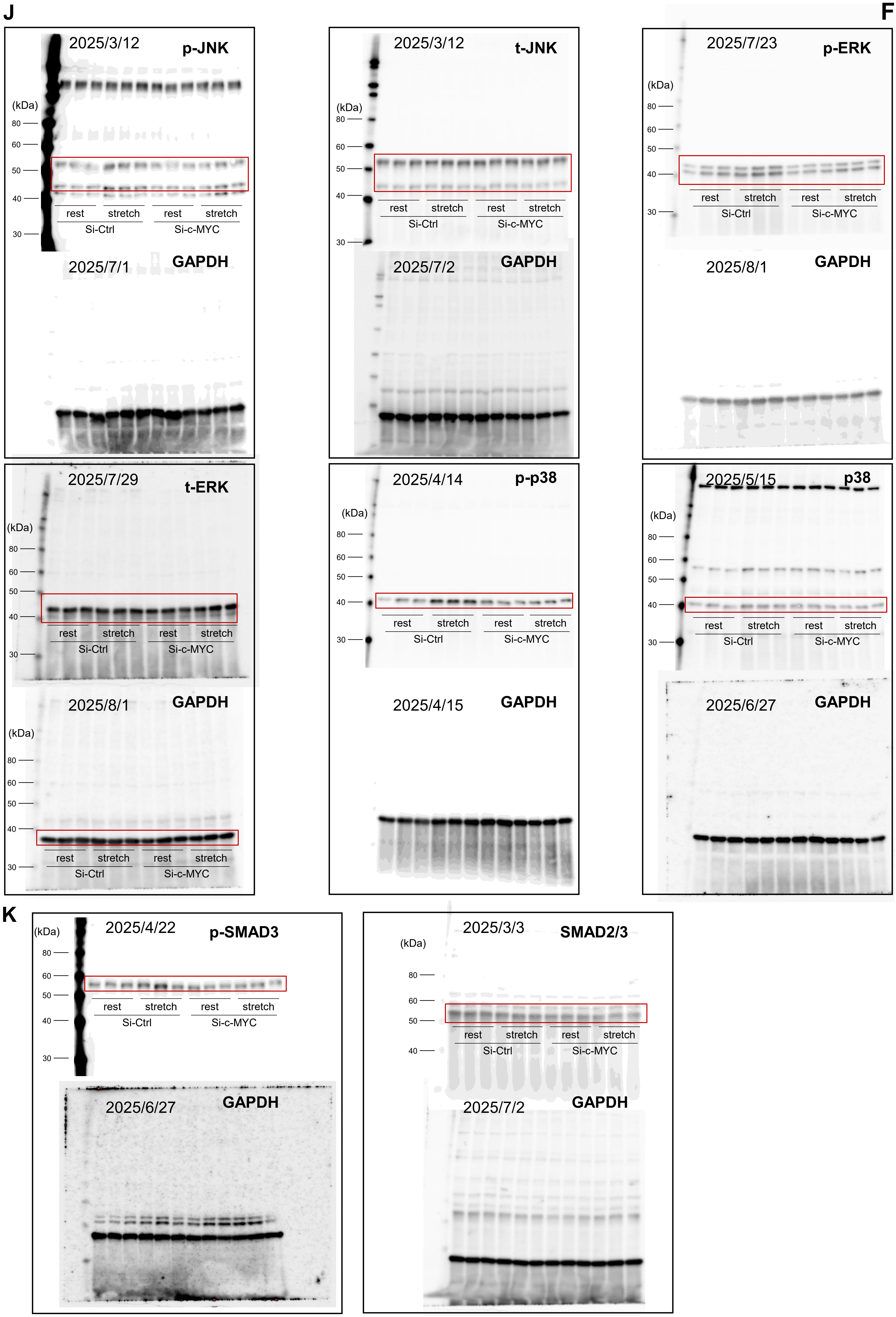
J
2025/3/12
p-JNK
(kDa)
80
60
50
40
30
rest
stretch
rest
stretch
Si-Ctrl
Si-c-MYC
2025/7/1
GAPDH
2025/3/12
t-JNK
2025/7/2
GAPDH
2025/7/23
p-ERK
2025/8/1
GAPDH
2025/7/29
t-ERK
2025/8/1
GAPDH
2025/4/14
p-p38
2025/4/15
GAPDH
2025/5/15
p38
2025/6/27
GAPDH
K
2025/4/22
p-SMAD3
2025/6/27
GAPDH
2025/3/3
SMAD2/3
2025/7/2
GAPDH

Figure 5

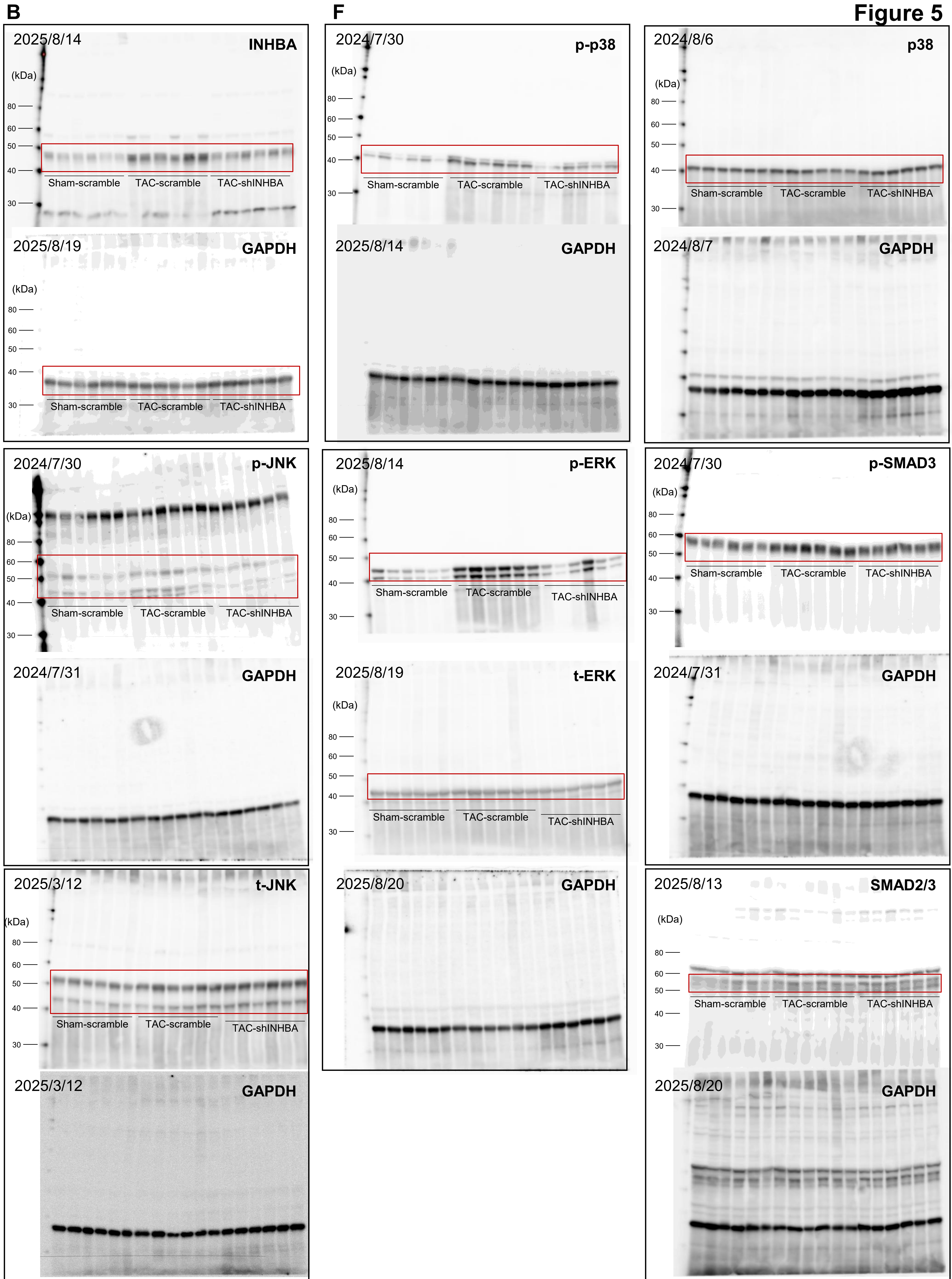
B
2025/8/14
INHBA
(kDa)
80
60
50
40
30
Sham-scramble
TAC-scramble
TAC-shINHBA
2025/8/19
GAPDH
2024/7/30
p-JNK
2024/7/31
GAPDH
2025/3/12
t-JNK
2025/3/12
GAPDH
F
2024/7/30
p-p38
2025/8/14
GAPDH
2025/8/14
p-ERK
2025/8/19
t-ERK
2025/8/20
GAPDH
2024/8/6
p38
2024/8/7
GAPDH
2024/7/30
p-SMAD3
2024/7/31
GAPDH
2025/8/13
SMAD2/3
2025/8/20
GAPDH

Figure 6

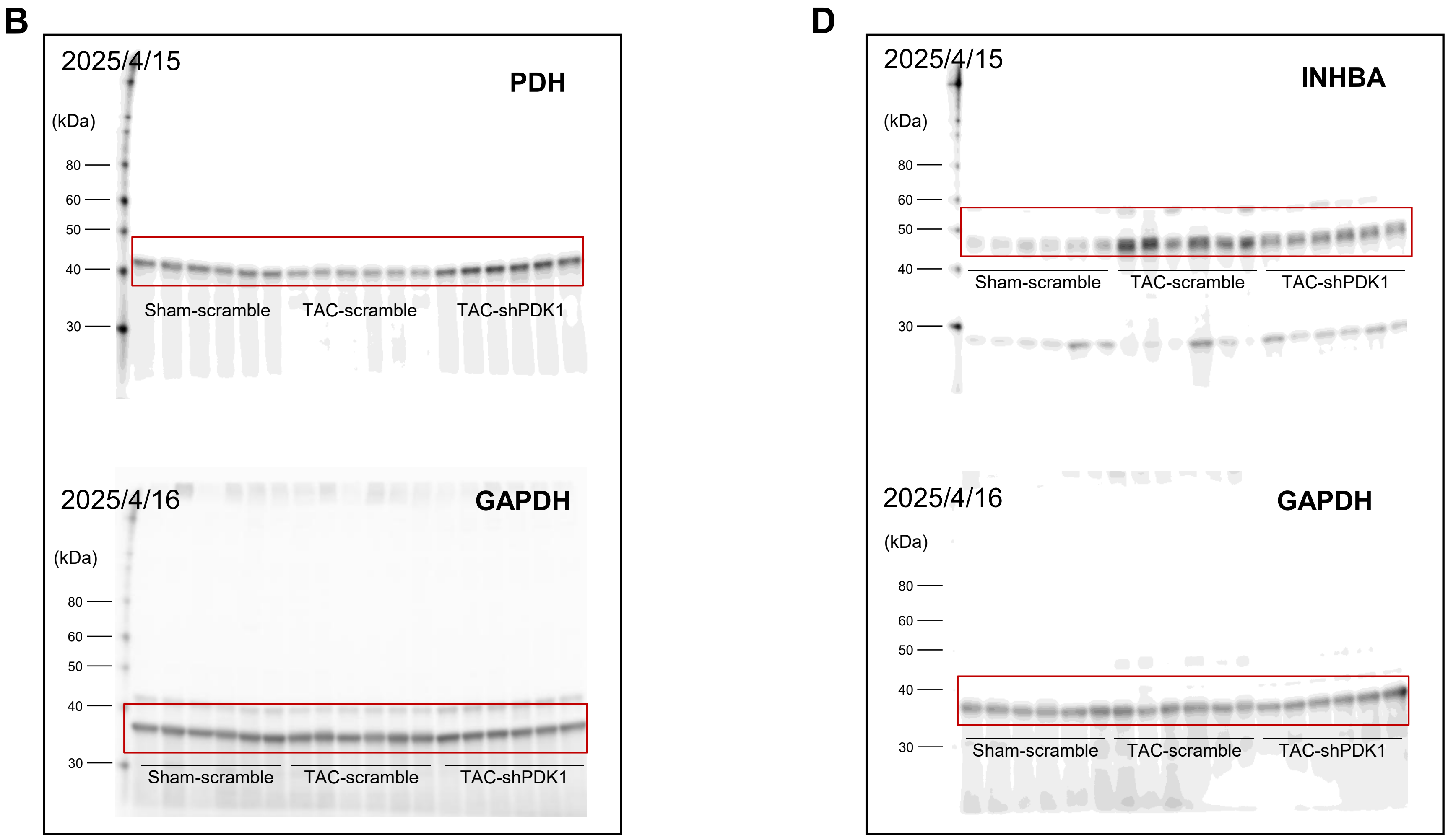
B
2025/4/15
PDH
(kDa)
80
60
50
40
30
Sham-scramble
TAC-scramble
TAC-shPDK1
2025/4/16
GAPDH
(kDa)
80
60
50
40
30
Sham-scramble
TAC-scramble
TAC-shPDK1
D
2025/4/15
INHBA
(kDa)
80
60
50
40
30
Sham-scramble
TAC-scramble
TAC-shPDK1
2025/4/16
GAPDH
(kDa)
80
60
50
40
30
Sham-scramble
TAC-scramble
TAC-shPDK1

Figure S4

C

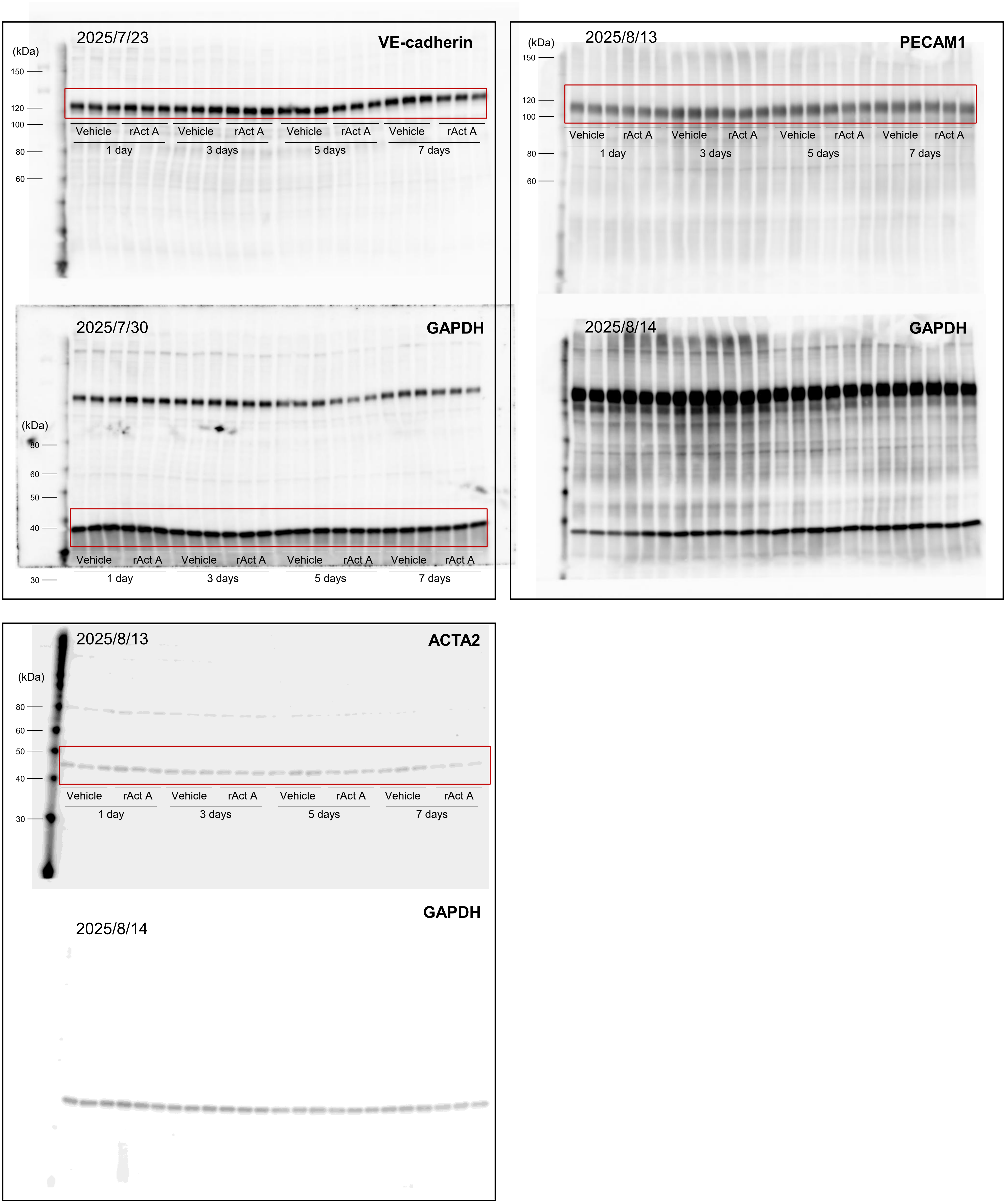

2025/7/23
VE-cadherin
(kDa)
150
120
100
80
60
Vehicle
rAct A
1 day
3 days
5 days
7 days
2025/7/30
GAPDH
(kDa)
80
60
50
40
30
2025/8/13
PECAM1
(kDa)
150
120
100
80
60
2025/8/14
GAPDH
2025/8/13
ACTA2
(kDa)
80
60
50
40
30
2025/8/14
GAPDH

A

Figure S6

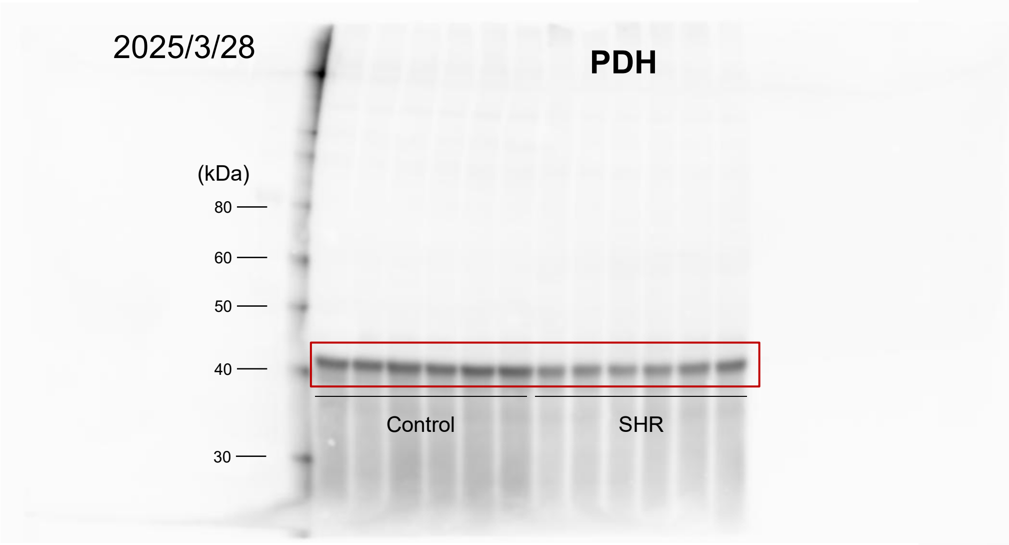

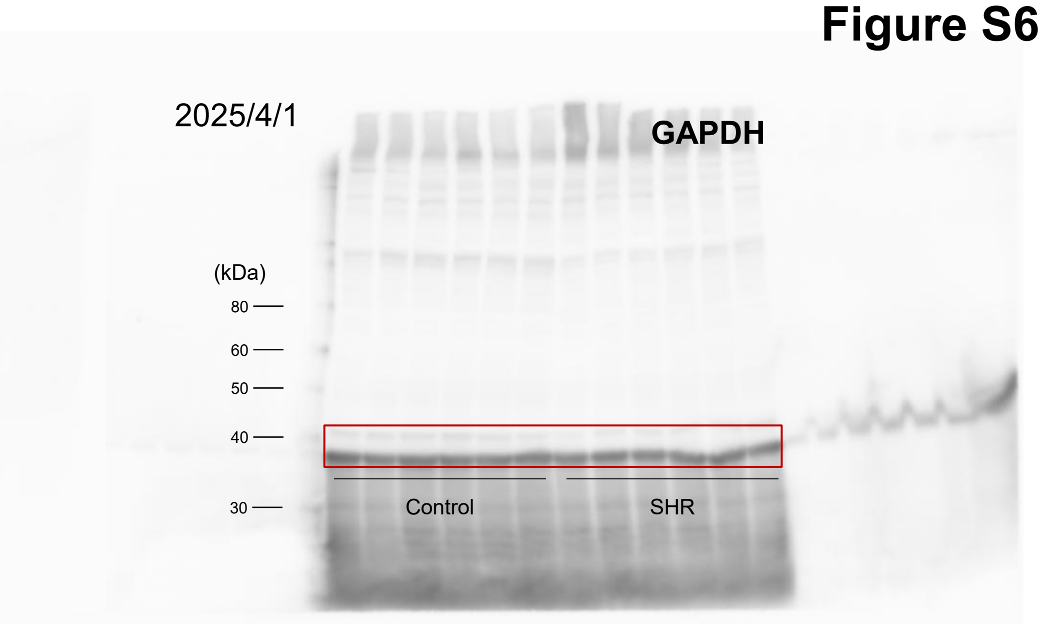

Due to membrane damage caused by stripping, the internal control could not be visualized.
